# Eltrombopag inhibits TET dioxygenase to contribute to hematopoietic stem cell expansion in aplastic anemia

**DOI:** 10.1172/JCI149856

**Published:** 2022-02-15

**Authors:** Yihong Guan, Metis Hasipek, Dongxu Jiang, Anand D. Tiwari, Dale R. Grabowski, Simona Pagliuca, Sunisa Kongkiatkamon, Bhumika Patel, Salendra Singh, Yvonne Parker, Thomas LaFramboise, Daniel Lindner, Mikkael A. Sekeres, Omar Y. Mian, Yogen Saunthararajah, Jaroslaw P. Maciejewski, Babal K. Jha

**Affiliations:** 1 Department of Translational Hematology and Oncology Research and; 2 Leukemia Program, Department of Hematologic Oncology and Blood Disorders, Taussig Cancer Institute, Cleveland Clinic, Cleveland, Ohio, USA.; 3 Department of Genetics and Genome Sciences, School of Medicine, Case Western Reserve University, Cleveland, Ohio, USA.; 4 Cleveland Clinic Lerner College of Medicine, Cleveland Clinic, Cleveland, Ohio, USA.; 5 Developmental Therapeutics, Case Comprehensive Cancer Center, Cleveland, Ohio, USA.

**Keywords:** Hematology, Clonal selection, Drug therapy, Hematopoietic stem cells

## Abstract

Eltrombopag, an FDA-approved non-peptidyl thrombopoietin receptor agonist, is clinically used for the treatment of aplastic anemia, a disease characterized by hematopoietic stem cell failure and pancytopenia, to improve platelet counts and stem cell function. Eltrombopag treatment results in a durable trilineage hematopoietic expansion in patients. Some of the eltrombopag hematopoietic activity has been attributed to its off-target effects, including iron chelation properties. However, the mechanism of action for its full spectrum of clinical effects is still poorly understood. Here, we report that eltrombopag bound to the TET2 catalytic domain and inhibited its dioxygenase activity, which was independent of its role as an iron chelator. The DNA demethylating enzyme TET2, essential for hematopoietic stem cell differentiation and lineage commitment, is frequently mutated in myeloid malignancies. Eltrombopag treatment expanded TET2-proficient normal hematopoietic stem and progenitor cells, in part because of its ability to mimic loss of TET2 with simultaneous thrombopoietin receptor activation. On the contrary, TET inhibition in TET2 mutant malignant myeloid cells prevented neoplastic clonal evolution in vitro and in vivo. This mechanism of action may offer a restorative therapeutic index and provide a scientific rationale to treat selected patients with TET2 mutant–associated or TET deficiency–associated myeloid malignancies.

## Graphical abstract

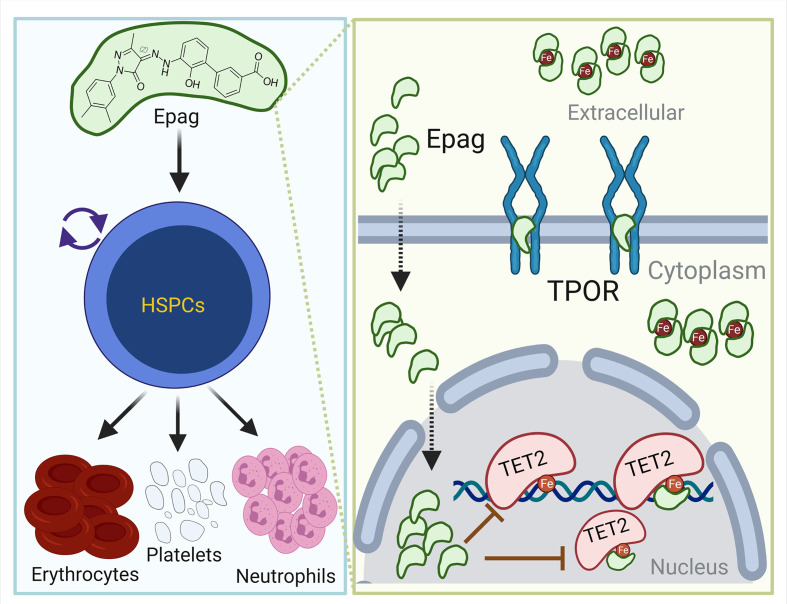

## Introduction

Idiopathic aplastic anemia is characterized by immune-mediated hematopoietic progenitor and stem cell (HSPC) destruction, resulting in deficiencies across all hematopoietic lineages (1–3). Despite the therapeutic successes of immunosuppressive therapies, approximately one-third of patients remain refractory (4), and many of the responses are incomplete. Moreover, some patients with aplastic anemia may experience clonal progression to myelodysplastic syndrome (MDS), associated with a poor prognosis (5–9). Recently, a synthetic thrombopoietin receptor (TPOR) agonist, eltrombopag (Epag), has been shown to be effective in aplastic anemia (10). In addition to the anticipated effect on platelet counts, Epag therapy also produced remarkable trilineage hematopoiesis (6, 11, 12). Subsequent studies confirmed the efficacy of Epag in de novo and refractory aplastic anemia (13) for increasing both the magnitude of response and the number of responders. These effects expanded the indication spectrum of Epag from immune thrombocytopenic purpura to aplastic anemia, establishing this drug as an essential hematologic therapeutic.

TPOR expressed on megakaryocytes typically signals via JAK/STAT pathway activation, but the presence of this receptor on early HSPCs could contribute to its stimulatory effects, driving the production of other blood cell lineages. However, Epag’s hematopoietic activity has been observed in murine models despite its inability to bind or activate murine TpoR. A similar finding has also been confirmed in TpoR-deficient mice (14–17) in which Epag treatment remarkably expanded HSPCs. These observations suggested that some of Epag’s activities may be TPOR independent, in contrast to peptide TPO analogs, e.g., romiplostim. These TPOR-independent effects of Epag were hypothesized to be due to its iron (III) chelating properties (14, 16, 17), but the molecular mechanism as to how this iron (III) binding could drive HSPC expansion remains speculative.

Epag’s effects on intracellular iron may affect certain iron-dependent epigenetic pathway(s) that promote HSPC self-replication. For instance, TET dioxygenases (TET1-3) are Fe^2+^- and α-ketoglutarate–dependent (αKG-dependent) DNA dioxygenases, which mediate CpG demethylation of promoters and enhancers in HSPCs. Consequently, by changing gene expression patterns, TETs control HSPC expansion and differentiation (18, 19). TET2 is the most abundant TET dioxygenase in HSPCs, and somatic loss-of-function (LOF) mutations of this gene frequently occur in myeloid neoplasia and clonal hematopoiesis of indeterminate potential, a prodromal condition in otherwise healthy elderly individuals associated with a higher rate of progression to leukemia compared with patients without hematopoiesis of indeterminate potential (20).

Here, we report the results of experiments designed to determine whether Epag affects the function of TET dioxygenases. Our findings clarified the mode of action of this drug in the HSPC compartment, specifically Epag’s activity in promoting multilineage clonal expansion and thus its therapeutic efficacy in aplastic anemia.

## Results

### The mechanisms of Epag action.

**Figure 1:**
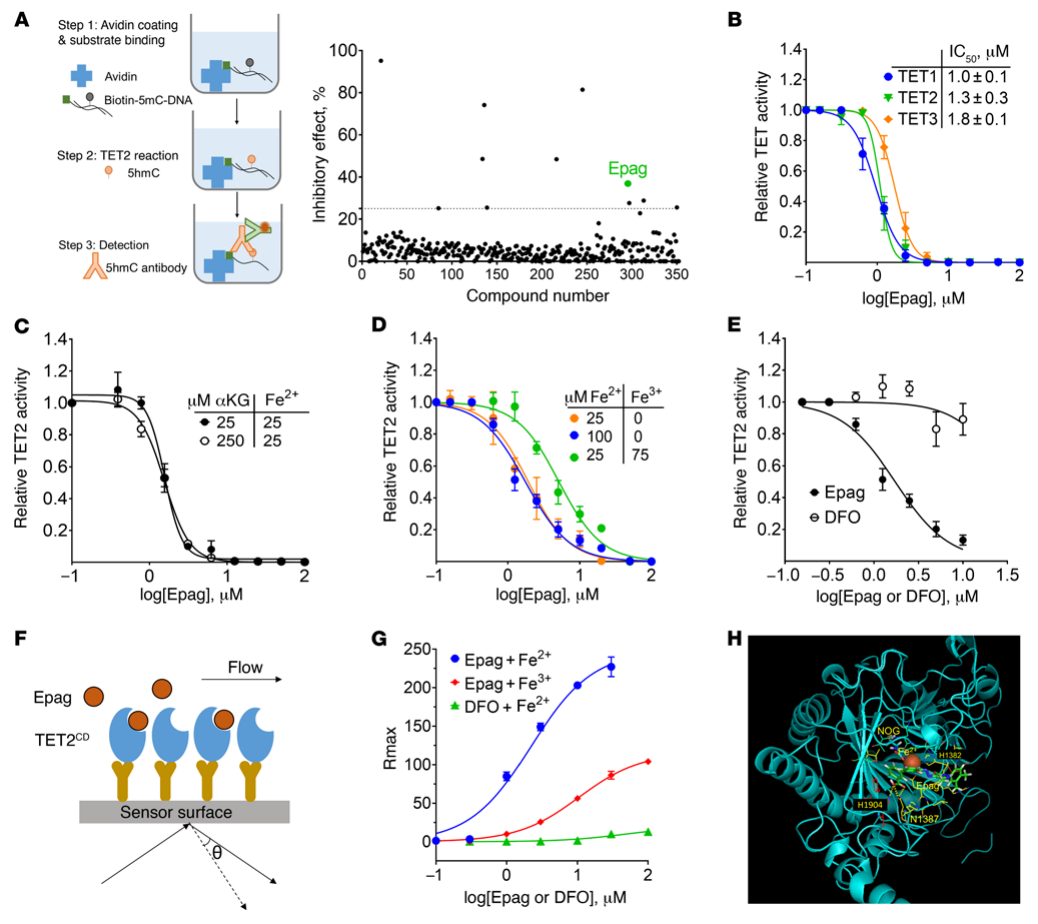
Epag binds and inhibits TET-dioxygenase activity in the presence of iron. (**A**) Epag as one of the top TET2 inhibitors identified by using an ELISA assay developed in-house. Schematic representation of ELISA assay for TET activity is on the left. (**B**) IC_50_ of Epag for TET1, TET2, and TET3 measured by ELISA. (**C** and **D**) TET2 inhibition by Epag cannot be restored by an excess amount of iron (II) or αKG but can only be partially restored by an excess amount of iron (III). (**E**) DFO does not inhibit TET2 activity. TET2 ELISA was performed with different concentrations of DFO and Epag. (**F**) Illustration of surface plasmon resonance assay to measure the binding affinity of Epag to TET2^CD^. The GST-TET2^CD^ was captured by anti-GST antibody on CM-5 sensor chip along with reference channel GST alone. (**G**) Equilibrium resonance maxima (R_max_) calculated by fitting the kon/koff using 1:1 Langmuir binding by BIAevaluation software and plotted; binding was measured in the presence of Fe^2+^ or Fe^3+^. (**H**) Epag mode of interaction with TET2-activated complex. In silico docking simulation of Epag with TET2. (**B**–**E** and **G**) Results are representative of 3 independent experiments performed and are expressed as mean ± SEM of at least 3 replicates.

Clinical and experimental observations suggest that a significant part of the hematopoietic activities of Epag is independent of its ability to activate TPOR-mediated signaling (14). During the search for TET-dioxygenase modulators performed using a cell-free high-throughput screen developed in-house employing a bioactive small-molecule library (LOPAC1280 and Selleck’s L1700; Figure 1A and Supplemental Figure 1A; supplemental material available online with this article; https://doi.org/10.1172/JCI149856DS1), Epag emerged as one of the most potent inhibitors of TET-dioxygenase activity targeting the TET2 catalytic domain (TET2^CD^). The IC_50_ for TET1 (1.0 ± 0.1 μM), TET2 (1.3 ± 0.3 μM), and TET3 (1.8 ± 0.1 μM) dioxygenases calculated from the dose-response curves suggest that Epag inhibited all 3 TET dioxygenases with a similar efficacy even in the presence of 25-fold molar excess of Fe^2+^ (Figure 1B). To test whether Epag is a competitive inhibitor of TET cofactors, we performed dose-dependent TET-dioxygenase inhibition in the presence of 25 and 250 μM of αKG or Fe^2+^. The IC_50_ of Epag under excess of Fe^2+^ or αKG remained unaffected (Figure 1C). The results indicate that Epag does not compete at the αKG binding site, since 10-fold molar excesses do not affect its ability to inhibit TET2 dioxygenase. The hematopoietic activity of Epag has been attributed to cellular and extracellular Fe^3+^ chelation (14, 17, 21, 22). Therefore, to probe the effect of free iron sequestration by Epag on its ability to inhibit TET activity, we preincubated excess Epag (25 μM) with varying concentrations of Fe^2+^ or Fe^3+^ and performed TET-dioxygenase activity assays. The addition of up to 8-fold molar excess of either form of iron (Fe^2+^ or Fe^3+^) did not rescue TET enzymatic activity (Supplemental Figure 1B). However, the addition of 75 μM of Fe^3+^ changed the IC_50_ of Epag from 1.3 μM to 5.1 μM (Figure 1D). This shift in the IC_50_ of Epag on TET inhibition was consistent with the proposed model of Epag sequestering free Fe^3+^. However, TET-dioxygenase inhibition by Epag is not fully attributable to its affinity for Fe^3+^ given that 100-fold molar excess of Fe^3+^ did not reverse TET-dioxygenase inhibition. In addition, to test whether iron chelators can act as broad-spectrum inhibitors of TET-dioxygenase activity, we tested deferoxamine (DFO) in similar conditions and found that iron chelation did not inhibit TET-dioxygenase activity (Figure 1E). These results suggest Epag’s effects on TET2 are independent of its ability to chelate Fe^3+^.

To further elucidate the specificity of Epag-mediated TET2 inhibition, we performed surface plasmon resonance–based (SPR-based) analysis to measure the direct binding to TET2^CD^ (Figure 1F). We observed a dose-dependent increase in the resonance response consistent with the direct binding of Epag to immobilized TET2 on a sensor chip (Figure 1G). However, we did not observe any significant affinity of Epag for TET2 in the absence of Fe^2+^. The addition of Fe^3+^ in place of Fe^2+^ significantly reduced the binding of Epag to TET2. DFO, a known broad-spectrum iron chelator, did not show TET2^CD^ binding or inhibitory activity (Figure 1G). This result further confirmed the notion that TET inhibition by Epag is not due to free iron chelation. To further understand the mode of binding of Epag to TET2, we performed in silico docking (AutoDock 4) running on AutoDock Tools (23) with the known crystal structure of TET2^CD^ (Protein Data Bank ID 4NM6) and interaction with its cofactors and substrates (24). Docking results indicated that Epag can form a tripartite complex with Fe^2+^ and TET2 (Figure 1H and Supplemental Figure 1D). The predictive model accounting for our binding data suggests a probable mode of Epag interaction with TET2 that incorporates His1382, Asp1387, and His1904 residues, while also engaging the catalytic site Fe^2+^ (Supplemental Figure 1, C and D). These amino acids are conserved among the family of TET dioxygenases, TET1 and TET3, and in their murine homologs (Supplemental Figure 1E).

### Epag diffuses into the nucleus.

**Figure 2:**
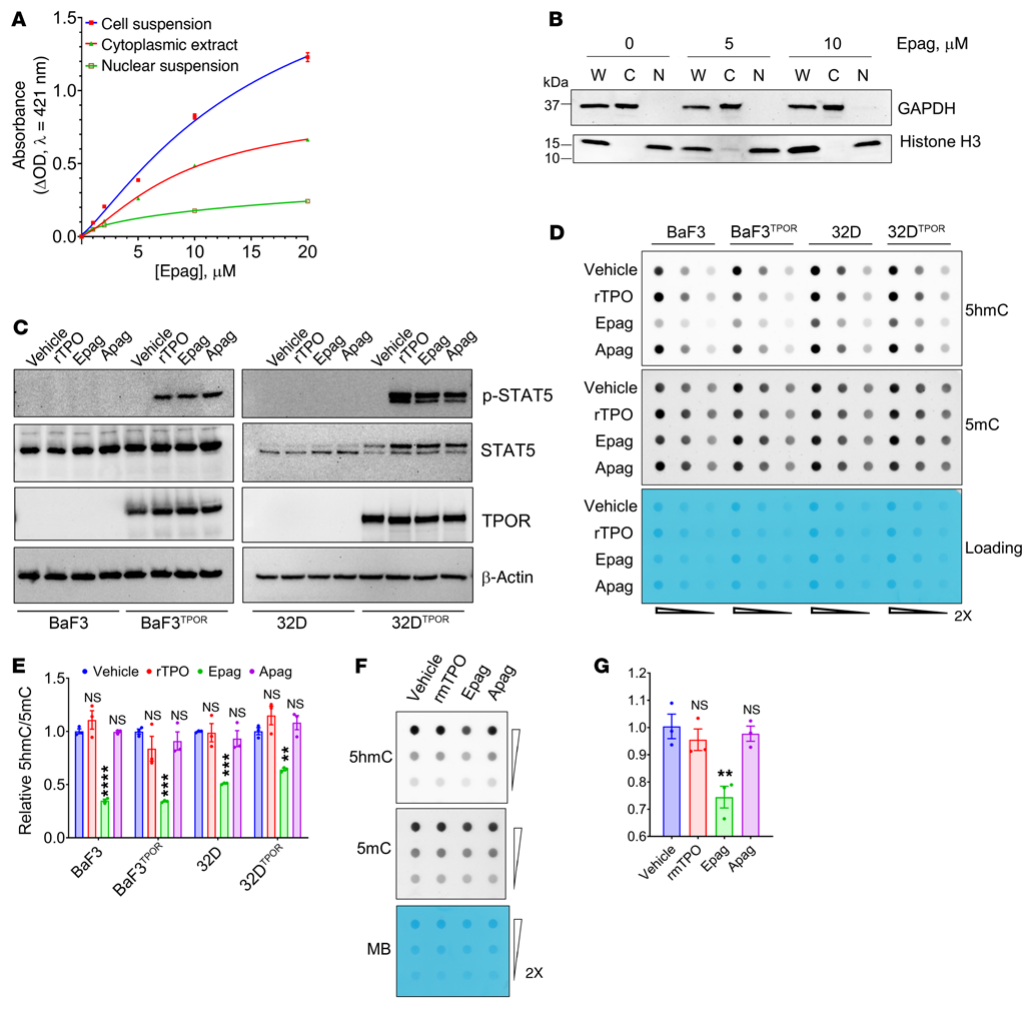
Epag inhibits TET dioxygenases in cells independent of TPOR. (**A**) Distribution of Epag into different subcellular compartments. 32D cells were treated with Epag for 30 minutes and washed and harvested. Cell suspensions, cytoplasmic extractions, and nuclear pellets were prepared. Absorbance at 421 nm was measured at a known concentration and plotted. The solid lines are best-fit curves in different fractions. (**B**) Western blot analysis of subcellular fractions of cells treated with Epag. W, whole-cell lysate; C, cytoplasmic fraction; N, nuclear fraction. (**C**) TPO-R activation by recombinant human TPO (rTPO), Epag, and Apag. Parental or human TPOR overexpressing BaF3 or 32D cells was treated with 100 ng/mL rTPO, 1 μM Epag, or 1 μM Apag for 30 minutes. Cells were washed and harvested for protein extraction followed by Western blot analysis. (**D** and **E**) After 30 minutes of treatment as described in **C**, cells were grown for additional 12 hours in complete media prior to genomic DNA extraction for 5hmC and 5mC quantification by dot blot. (**F** and **G**) Epag inhibits TET activity in mouse bone marrow mononuclear cells. Murine bone marrow mononuclear cells were treated with 100 ng/mL recombinant murine TPO (rmTPO), 1 μM Epag or 1 μM Apag as in panels **C** and **D,** and 5hmC and 5mC were quantified by dot blot. (**A**–**D** and **F**) Results are representative of 3 independent experiments performed. (**E** and **G**) Data are expressed as mean ± SEM of 3 replicates. **P < 0.01, ***P < 0.001, ****P < 0.0001, and NS (*P* > 0.05) by 1-way ANOVA with Dunnett’s test of indicated treatment group and the vehicle control.

There have been several reports characterizing Epag’s chemical properties, but its intracellular functions have not been systematically studied (25, 26). We analyzed the UV-visible spectroscopic properties of Epag by determining the absorption maxima in aqueous buffers at different pH levels; absorption maxima remained unchanged at 421 nm in the presence and absence of fractionated subcellular suspensions (Supplemental Figure 2, A and B). By assaying the standard dose-dependent increase in absorption (observed up to 80 μM; Supplemental Figure 2B), we estimated the fraction of Epag present in the nucleus. We found that nearly 24% of Epag was partitioned into the nuclear fraction within 30 minutes of exposure, indicating it can diffuse into the nucleus and thus may be accessible to TET2 located in the nucleus (Figure 2A and Supplemental Figure 2C). The purities of the cytoplasmic and nuclear fractions were analyzed by Western blot using the specific markers GAPDH (cytoplasmic fraction) and histone H3 (nuclear fraction). The result demonstrated that both fractions used in the study for Epag partitioning were pure and there was no detectable cross-contamination (Figure 2B; see complete unedited blots in the supplemental material).

### Epag inhibits TET activity independent of TPOR activation.

The known functions of Epag have been attributed primarily to its agonistic effect on TPOR. More broad-spectrum effects on hematopoiesis have been postulated to be due to its ability to chelate cellular and extracellular iron (III) (14, 16, 17). To test whether Epag-induced TpoR signaling affects TET activity, we engineered murine cell lines BaF3 and 32D to express human TPOR. Irrespective of the presence or absence of TPOR, Epag inhibited TET-dioxygenase activity as indicated by the decrease in 5hmC, a reliable measure of cellular TET-dioxygenase activity (Figure 2, C–E; see complete unedited blots in the supplemental material). This effect was irrespective of JAK/STAT pathway activation (mediated by TPO-R activation) in that STAT5 phosphorylation levels showed no association with the magnitude of TET inhibition by Epag (Figure 2, C–E). Similarly, the presence of TPOR signaling activation did not contribute to TET inhibition by Epag in human TPOR overexpressing BaF3 and 32D (Figure 2, C–E). While TPO, Epag, and avatrombopag (Apag; a chemically unrelated non-peptidyl TPOR agonist) all activated the JAK/STAT pathway to the same extent in these cells, only Epag exhibited a robust TET-inhibitory effect reflected in the global reduction of 5hmC (Figure 2, C–E). Next, primary murine bone marrow mononuclear cells were treated with vehicle, TPO, Epag, or Apag, and we observed that only Epag treatment reduced 5hmC compared with vehicle (Figure 2, F and G). Thus, Epag-mediated TET inhibition in these cells was specific and independent of the presence or absence of TPOR signaling.

### Epag treatment phenocopies loss of Tet2.

**Figure 3:**
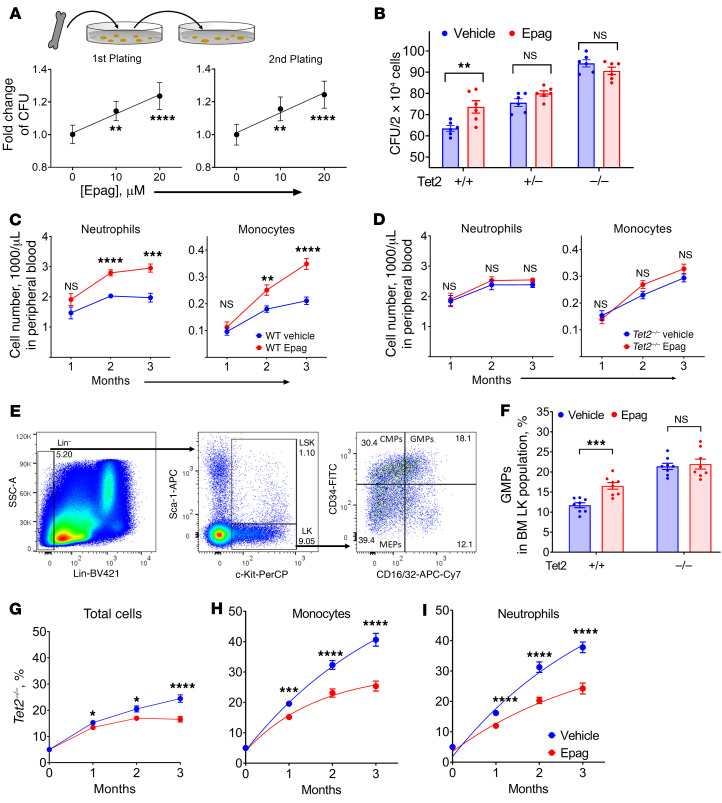
Epag treatment mimics loss of Tet2, and expansion of myeloid compartment is Tet2 dependent. (**A**) Epag dose response in the CFUs of *Tet2^+/+^* murine bone marrow cells. (**B**) Epag increased CFUs in *Tet2^+/+^* but not *Tet2^+/–^* and *Tet2^–/–^* cells. Data of the second plating are shown. (**A** and **B**) Six mice per group/treatment were used in 2 independent experiments. (**C** and **D**) Epag significantly increased neutrophil and monocyte count in vivo in *Tet2^+/+^* but not in *Tet2^–/–^* graft recipient mice. *Tet2^+/+^* CD45.1, Pep Boy mice were lethally irradiated prior to the transplant of 2 million *Tet2^+/+^* or *Tet2^–/–^* bone marrow cells (CD45.2) via tail vein injection. Peripheral blood samples were counted by Hemavet. (**E**) Gating strategy of flow cytometry analysis for HSPCs, Lin^–^Sca-1^+^c-Kit^+^ (LSK), Lin^–^Sca-1^–^c-Kit^+^CD34^+^CD16/32^–^ (CMPs), Lin^–^Sca-1^–^c-Kit^+^CD34^+^CD16/32^+^ (GMPs), and Lin^–^Sca-1^–^c-Kit^+^CD34^–^CD16/32^–^ (MEPs). (**F**) Epag increased the percentage of GMPs in *Tet2^+/+^* but not *Tet2^–/–^* grafted mice. (**G**–**I**) Percentage of *Tet2^–/–^* cells in indicated populations in bone marrow of transplanted mice. PEP mice were lethally irradiated and received 2 million bone marrow cells consisting of 95% *Tet2^+/+^* (PEP, CD45.1) and 5% *Tet2^–/–^* (CD45.2) through tail vein injection. Blood was harvested for flow cytometry analysis, CD11b^+^CD11c^–^Ly6C^+^Ly6G^–^ (monocytes), and CD11b^+^CD11c^–^Ly6C^lo^Ly6G^+^ (neutrophils). The antibodies used were FITC-CD45.1, PE-CD11c, APC-Ly6C, Apc-Cy7-Ly6G, and PerCP-CD11b. Data are representative of experiments done twice. (**C**, **D**, and **F**–**I**) Mice were randomly divided into 2 groups and either treated with 50 mg/kg Epag or vehicle (water) by oral gavage. A total of 4 donor mice and 8 recipient mice were used per group in 2 independent experiments. Data are expressed as mean ± SEM of 8 replicates. *P < 0.05, **P < 0.01, ***P < 0.001, ****P < 0.0001, and NS (*P* > 0.05) by 1-way ANOVA using Dunnett’s test (**A**) and 2-tailed unpaired *t* test (**B**–**D** and **F**–**I**).

Studies of the consequences of *TET2* mutations in MDS and murine models have demonstrated that loss of *TET2* contributes to HSPC expansion and myeloproliferation (27–30). Consistent with this, we observed that Epag treatment significantly increased the colony-forming ability of murine HSPCs in vitro (Figure 3A and Supplemental Figure 4, A and B). Since Epag treatment mimics LOF of *Tet2*, we used *Tet2^–/–^* mice as a control to investigate whether Epag’s effect is indeed *Tet2* dependent. Consistent with cell-free and suspension cell culture studies, colony-forming assays showed that Epag treatment increased the colony formation in *Tet2^+/+^*; in particular, there were significant increases in CFU-G, CFU-M, and CFU-GM, whereas no significant effect was observed in the CFUs of *Tet2^+/–^ or Tet2^–/–^* murine HSPCs (Figure 3B and Supplemental Figure 3A). To recapitulate this effect in vivo, we performed C57BL/6J-CD45.2 *Tet2^+/+^* and *Tet2^–/–^* syngeneic bone marrow transplant in CD45.1 Pep Boy mice lethally irradiated with 9.6 Gy radiation followed by Epag treatment (Supplemental Figure 3A). In these single syngeneic bone marrow grafts, Epag treatment significantly increased monocyte and neutrophil counts (Figure 3C and Supplemental Figure 4C) with no observable change in RBCs, platelets, WBCs, or lymphocyte counts of WT graft recipient mice treated with Epag (Supplemental Figure 3B). Consistent with the results of colony-forming assays, the effect of Epag on neutrophils and monocytes was not observed in *Tet2^–/–^* graft recipient mice (Figure 3D, Supplemental Figure 3C, and Supplemental Figure 4, D and E). This observation suggested that the effect of Epag observed in murine hematopoiesis is predominantly due to its inhibitory effects on Tet2. Analysis of the bone marrow from *Tet2^+/+^* and *Tet2^–/–^* graft recipient CD45.2 Pep Boy mice after 3 months of treatment at euthanization showed that Epag significantly expanded CD34^+^CD16/32^+^lineage^−^Sca-1^−^Kit^+^ granulocyte–macrophage progenitors (GMPs) along with a small but significant increase in the lineage^−^Sca−1^+^c-Kit^+^ stem cell (LSK) population (Figure 3, E and F, Supplemental Figure 3D, and Supplemental Figure 4). On the contrary, Epag treatment did not affect the blood or bone marrow composition of *Tet2^–/–^* graft recipient mice (Figure 3F and Supplemental Figure 3D) compared with the vehicle control group (Figure 3F, Supplemental Figure 3D, and Supplemental Figure 4).

### Epag treatment restricts the clonal evolution of Tet2^–/–^ in vivo.

Since Epag treatment expands *Tet2^+/+^* but not *Tet2^–/–^* cells, we tested whether the expansion of WT cells can be used to restrict the clonal evolution of *Tet2^–/–^* cells in vivo using competitive bone marrow transplant in a murine model system. For this purpose, we used lethally irradiated CD45.1 Pep Boy mice as recipients and transplanted them with 2 million chimeric donor bone marrow cells composed of 95% CD45.1 Pep Boy (*Tet2^+/+^*) and 5% CD45.2 C57BL/6J (*Tet2^–/–^*; Supplemental Figure 3E). Once the graft was established (2 weeks after transplant), the mice were randomly divided into 2 groups with 1 receiving Epag (50 mg/Kg, p.o.; 5 days/week). The evolution of blood chimerism over time was monitored using the surface markers CD45.1, CD45.2, or both (Supplemental Figure 3F). Consistent with the previous reports, *Tet2^–/–^* cells expanded rapidly in the control group compared with WT cells (27–29). Interestingly, Epag treatment significantly slowed the clonal evolution of *Tet2^–/–^* cells compared with controls (Figure 3G). At the end of the treatment, there was a 26% reduction in the *Tet2^–/–^* fraction compared with the control. Further analysis of different subpopulations of leukocytes demonstrated a bigger difference in monocytes (CD11b^+^CD11c^–^ Ly6C^+^Ly6G^–^) and neutrophils (CD11b^+^CD11c^–^Ly6C^lo^Ly6G^+^) compared with the control. After 3 months of Epag treatment, the *Tet2^–/–^* fractions of monocytes and neutrophils were nearly half of the control (Figure 3, H and I, and Supplemental Figure 3E). We did not observe any change in CD4^+^, CD8^+^, or B220^+^ cells in the treatment group compared with controls (Supplemental Figure 3G and Supplemental Figure 4, F and H).

The chimerism of different subpopulations of bone marrow cells at the time of euthanization was analyzed using CD45.1 and CD45.2 surface markers along with lineage-specific markers for LSKs (Lin^–^Sca-1^+^c-Kit^+^, stem cell), LKs (Lin^–^Sca-1^–^c-Kit^+^), GMPs (CD34^+^CD16/32^+^Lin^–^Sca-1^–^Kit^+^), CMPs (Lin^–^Sca-1^–^c-Kit^+^CD34^+^CD16/32^–^), and MEPs (Lin^–^Sca-1^–^c-Kit^+^CD34^–^CD16/32^–^) (Supplemental Figure 3H). Epag treatment reduced the rate of clonal evolution of *Tet2^–/–^* cells in the bone marrow at the time of euthanization, as observed in total *Tet2^–/–^* cell fraction in the bone marrow. A consistent decrease in LSK, LK, GMP, CMP, and MEP fractions was observed; however, the difference did not reach the level of statistical significance (Supplemental Figure 3H and Supplemental Figure 4G).

### Epag treatment inhibits TET dioxygenase in primary human hematopoietic cells.

**Figure 4:**
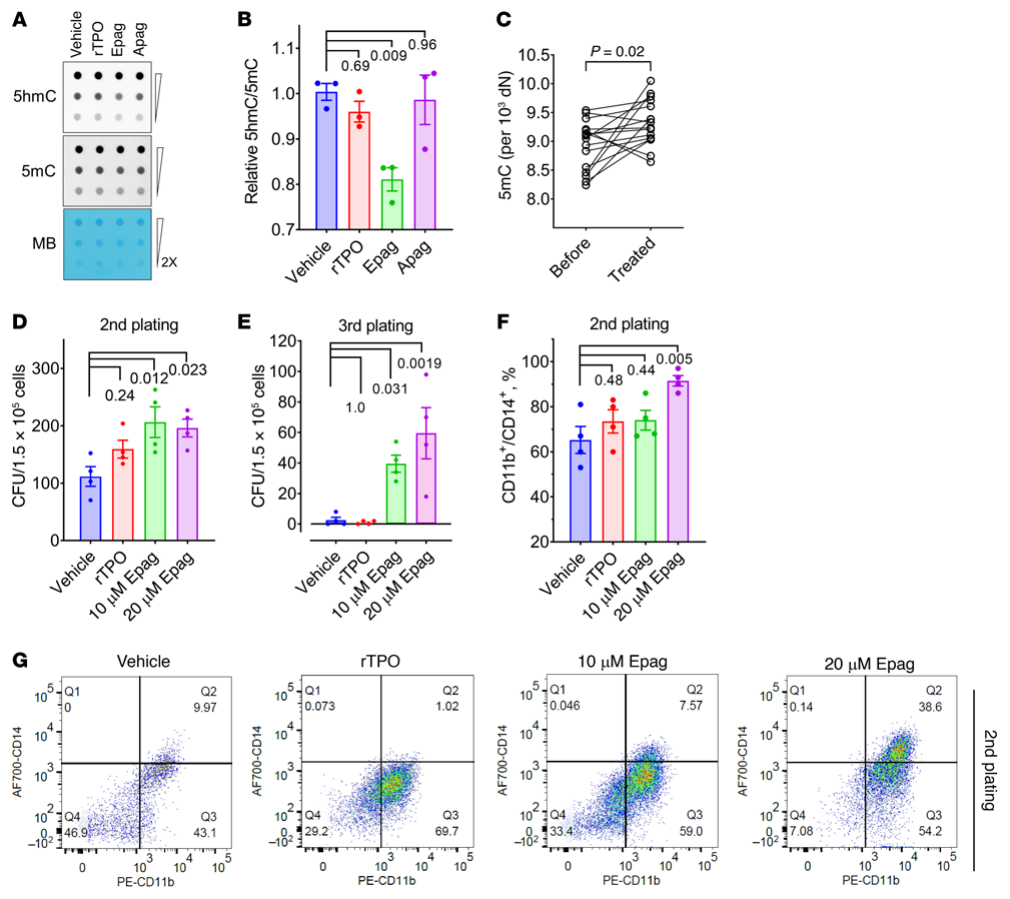
Epag inhibits TET activity in humans. (**A** and **B**) Epag inhibits TET activity in human bone marrow mononuclear cells. Cells were treated with 100 ng/mL rTPO, 1 μM Epag, or 1 μM Apag for 30 minutes and followed by culturing for additional 12 hours with 10% FBS and 100 μM ascorbic acid. Cells were washed and harvested for DNA extraction and used for dot blot analysis for 5hmC and 5mC. **B** is the quantification and analysis of results in **A**. (**C**) Epag inhibits TET activity in patients. Peripheral blood mononuclear cells from patients with aplastic anemia were isolated before and in the middle of taking Epag. Genomic DNA was extracted for mass spectrometer analysis. Taking Epag significantly increased the global 5mC level, indicating TET inhibition by Epag. (**D** and **E**) Epag increases CFUs in human bone marrow mononuclear cells from healthy donors (NBMs), *n =* 4. Mononuclear cells were seeded in Methocult with indicated concentrations of Epag or 100 ng/mL rTPO. CFUs were counted after the second and third plating. (**F** and **G**) Follow-up analysis of cells harvested from the second plating of colony-forming assay in **D**. (**A** and **G**) Results are representative of at least 3 independent experiments performed. Data are expressed as mean ± SEM of 3 (**B**) or 4 (**D**–**F**) biological replicates in 3 independent experiments. *P* values from Dunnett’s test are indicated.

Epag treatment transiently mimics the loss of TET2 independent of TpoR activation in vitro in murine cells and in vivo transplant models (Figure 3). To test whether TET inhibition in human cells is also TPOR independent, we used primary human bone marrow mononuclear cells derived from healthy donors and treated them with TPO, Epag, Apag, and vehicle alone as a control. Only Epag treatment reduced 5hmC; neither TPO nor Apag had any effect on global 5hmC, a marker for TET activity in primary bone marrow cells (Figure 4, A and B).

The TET-inhibitory effect of Epag was further evident by the hypermethylation of the genomic DNA isolated from PBMCs from patients with aplastic anemia (*n =* 16) after Epag treatment. Global 5mC content measured using liquid chromatography with tandem mass spectrometry (LC/MS/MS) demonstrated a significant increase in 5mC content after Epag treatment compared with the PBMCs isolated before treatment (Figure 4C).

To further understand the consequences of TET inhibition by Epag in HSPCs, we used a serial replating colony-forming assay using human Methocult. Bone marrow cells from 4 healthy donors were treated with recombinant human TPO (rTPO) or Epag in addition to the standard cocktail of growth factors (stem cell factor, IL-3, IL-6, erythropoietin, G-CSF, and GM-CSF). Epag treatment significantly prolonged the clonogenic potential of healthy bone marrow cells compared with control rTPO treatment as observed in the second and third plating (Figure 4, D and E, and Supplemental Figure 5A). Interestingly, in the third plating, no colony was observed in the control or rTPO treatment, while the Epag-treated group retained significant colony-forming capacity (Figure 4E). The flow cytometric analysis of cells after the second and third plating showed that a higher proportion of cells (86%–97%) in Epag-treated cultures expressed CD11b/CD14 compared with control or rTPO groups (Figure 4, F and G, and Supplemental Figure 5, B–E). These results suggest that the effect of Epag may be independent of *TPOR* status, instead mimicking TET2 loss reflected in increased fractions of CD11b^+^/CD14^+^ cells (31).

### Epag treatment prevents the clonal growth TET2^MT^ cells.

**Figure 5:**
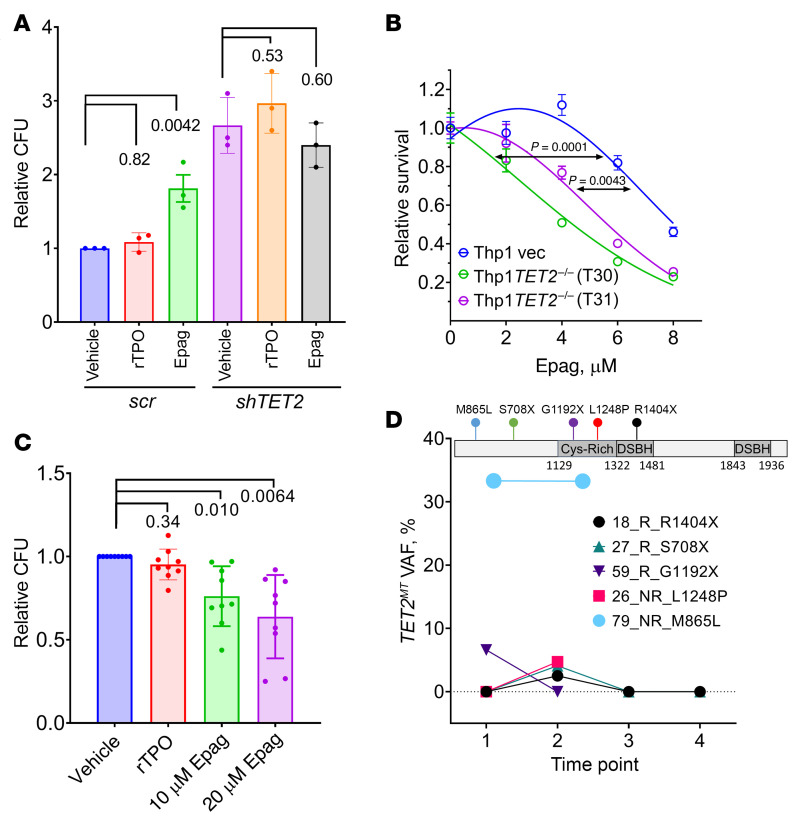
Epag treatment restricts the clonal growth of *TET2^MT^* malignant cells. (**A**) Epag increased CFUs of HSPCs depending on its TET2 inhibitory but not TPOR activation function. CD34^+^ cells were purified from cord blood samples (*n =* 3) and then infected with lentivirus with TET2 or scrambled shRNA. Cells were plated in Methocult 2 days after infection in the presence of puromycin and 10 μM Epag or 100 ng/mL rTPO. Relative CFUs presented are after the third plating. (**B**) *TET2^–/–^* cells are more sensitive to Epag treatment. TET2 mutant cells (T30 and T31), as well as vector control cells (vec), were treated with different concentrations of Epag for 3 days. Cell survival was calculated by CellTiter-Glo assay. The AUCs were calculated and used for statistical analysis. (**C**) Epag significantly restricted the clonal growth of *TET2^MT^* malignant cells. Mononuclear cells were purified from myeloid neoplasm patient bone marrow (*n =* 9) with TET2 mutations. Mononuclear cells were seeded in Methocult with indicated concentrations of Epag or 100 ng/mL rTPO. CFUs were counted after second plating, and relative colony numbers were plotted. (**D**) TET2 mutation variant allele frequency (VAF) of 5 patients with aplastic anemia who received Epag treatment (33). R, responder; NR, nonresponder. Time points: 1, baseline before Epag initiation; 2, primary end point (24 weeks); 3, longest time point available on Epag; 4, longest time point available off Epag after achieving robust response. (**B**) Results are representative of 3 independent experiments performed. Data are expressed as mean ± SEM of 3 (**A**) or 9 (**C**) biological replicates in 3 independent experiments. *P* values are from 1-way ANOVA with Dunnett’s test.

To further confirm the role of TET2 inhibition in Epag’s hematopoietic activity and evaluate its impact on TET2-deficient cells, we performed *TET2* knockdown in CD34^+^ cells derived from human cord blood using lentiviral TET2 targeting shRNA along with scrambled shRNA (*scr*) control as described earlier (31). Consistent with prior reports (29, 31), knockdown of *TET2* in CD34^+^ cells resulted in a nearly 3-fold increase in colony numbers compared with *scr* shRNA control. Consistent with the murine model, the Epag treatment of human CD34^+^, cells transduced with *scr* shRNA increased colonies by nearly 2-fold compared with the vehicle treatment; this increase was not observed in *TET2^KD^* CD34^+^ cells (Figure 5A). Interestingly, a consistent decrease in colony number (statistically nonsignificant) in the TET2 knockdown cells was observed in the Epag treatment group. rTPO treatment had no significant effect on the colony numbers (Figure 5A).

Recent reports suggest that highly proliferative myeloid leukemia cells are critically dependent on TET3 (32), particularly in the absence of TET2 (23). To test whether TET inhibition by Epag imposes growth restrictions, particularly on TET2 knockout myeloid leukemia cells compared with WT cells, we tested isogenic THP1*TET2^KO^* cells generated using CRISPR-Cas9 and treated with increasing doses of Epag (Figure 5B). *TET2^–/–^* THP1 cells have a higher sensitivity to Epag compared with the nontargeting gRNA THP1 control cells, suggesting that the inhibition of residual TET activity mostly coming from TET3 may be detrimental for TET2 mutant leukemia cells (32). Thus, while Epag inhibits TET2 in TET dioxygenase–proficient bone marrow cells from patients with aplastic anemia, leading to HSPC expansion, in TET2-deficient leukemia cells it may restrict their growth because of their dependence on TET3 for demethylation of promoters and enhancers of survival and proliferative genes. This in vitro cell-line model was further confirmed in primary cells derived from patients with TET2 mutant myeloid neoplasms. The effect of Epag on TET2 mutant myeloid neoplasm patient-derived mononuclear cells was tested in 9 patients (5 with biallelic and 4 with monoallelic inactivation of TET2) using colony-forming assays in the presence or absence of Epag (Figure 5C, Supplemental Figure 6, and Supplemental Table 1). Treatment of Epag led to a nearly 2-fold reduction in the colony numbers compared with vehicle treatment (Figure 5C and Supplemental Figure 6). The effect was more pronounced in the second plating (Figure 5C). We did not observe any statistically significant correlation of Epag treatment with variant allele frequency (VAF) or the monoallelic or biallelic inactivation under in vitro culture conditions (Supplemental Figure 6, D and E).

Since Epag has been extensively studied in clinical trials and some of the clinical trials have patient mutation data available, we investigated those details and analyzed the publicly available data. In a study with a cohort of 43 aplastic anemia patients by Winkler et al. (33), there were 5 patients with TET2 mutations at different points in Epag treatment, among which 3 were LOF truncating mutations and 2 missense alterations of unclear significance (Figure 5D). Interestingly, all 3 truncating TET2 mutants were responders, and these clones completely disappeared at the end of the treatment, suggesting that Epag may have a restrictive effect on otherwise proliferative *TET2^MT^* clones. In contrast, 2 patients had missense mutations (M865L and L1248P) considered inconsequential for TET2 enzymatic function (Figure 5D). These patients were nonresponders, and TET2 mutant clone size did not change in these cases. The results seen in the responders with pathogenic mutations agree with our hypothesis that *TET2^MT^* responding to Epag mediated TET inhibition due to a proliferative advantage of WT HSPCs, thus competitively restricting and preventing the clonal evolution of *TET2^MT^* cells as well as preventing the otherwise proliferative dominant clone in certain patients.

## Discussion

Newly discovered biologic activities of drugs with seemingly established specificity often lead to new indications, sometimes years after the initial FDA approval. The remarkable efficacy of Epag in aplastic anemia, a prototypic hematopoietic stem cell deficiency disorder, suggests that it may have alternative activity in addition to its known effects on megakaryocytopoiesis. Earlier, it was reported that some of Epag’s TPOR-independent hematopoietic activities are due to intracellular and extracellular iron (III) chelation (14, 16, 17, 21). However, a large part of Epag’s mechanisms of action remained unclear. Here, using a series of in vitro cell-free and cell-based assays along with in vivo small animal models, we report that parts of Epag’s TPOR-independent clinical activities may be due to its ability to inhibit TET dioxygenase by direct interaction with the catalytic domain. This reversible and transient inhibition to some extent phenocopies the hematopoietic consequences of TET2 LOF observed in *TET2* mutant cells, i.e., expansion of HSPCs and myeloid skewing.

Our results showed that while Fe^2+^ is required for Epag binding to TET2, its inhibitory effect is not due to sequestration of intracellular or extracellular iron and cannot be rescued by adding excess iron. Thus, the following 2 plausible reactions can be envisioned to explain the TET-inhibitory activity of Epag: (a) *TET2 +* *Fe^2+^ + Epag ↔* [*Fe^2+^*-*Epag*] *+* [*TET2*] or (b) *TET2 + Fe^2+^ + Epag ↔* [*TET2*-*Fe^2+^-Epag*].

The addition of several-fold molar excess α-KG failed to overcome the inhibitory effect of Epag, suggesting that it is not a competitive inhibitor of α-KG. Further analysis of direct binding kinetics using SPR confirmed the specificity of Epag interaction with TET2^CD^ in the presence of Fe^2+^. Substitution of Fe^2+^ by Fe^3+^ in the binding buffer significantly reduced Epag affinity to TET2^CD^. Interestingly, 750-fold molar excess Fe^3+^ shifted 2-fold IC_50_, suggesting that Epag chelates Fe^3+^ with the caveat that its affinity for TET2^CD^ is much higher than that for Fe^3+^. DFO, a known powerful iron chelator, showed no significant binding to TET2, suggesting that Epag’s interaction with the TET2 catalytic domain is specific and requires Fe^2+^ as a cofactor. Using a cell-free in vitro model system coupled with in silico molecular docking, we demonstrated that Epag directly binds to TET2^CD^ via Fe^2+^ and potently inhibits its dioxygenase function in physiologically relevant doses (34). Therefore, the experimental evidence presented here supports a model where Epag forms a specific tripartite complex [*TET2-Fe^2+^-Epag*], which traps the catalytic site in an inactive conformation following equation (b) above.

Epag’s effect on TET2 in cell-free systems is clinically as well as biologically relevant since it is partitioned into the nucleus within half an hour to exert its inhibitory activity on TET dioxygenases in cells, as reflected in the reduced genomic 5hmC content. Significant effects of Epag have been observed with 50 mg and 75 mg daily doses in humans (10, 35). Pharmacokinetic characterization of healthy volunteers and patients showed that peak plasma concentration at steady state (Cmax) reaches around 8.0 and 12.7 μg/mL after once-daily oral administration of 50 and 75 mg, respectively (34, 35). Given the Epag molecular weight of 442.5 g/mol, the Cmax is around 10 μM or 20 μM for a 50 mg or 75 mg oral dose. In the present study, we observed an IC_50_ of approximately 1 μM for TET2 inhibition, which is more than one-tenth of the physiological concentration achieved for Epag in patients. Consistent with physiological steady-state Cmax, we observed a significant effect of Epag at 10 μM and 20 μM across different cellular assays.

The effect of Epag treatment on DNA dioxygenase activity was independent of TPOR signaling. Epag is known to be human TPOR specific with no known activation of murine JAK/STAT signaling (14, 25, 26), but the effect on TET2 was observed in murine hematopoietic cells in the absence of TpoR activation. Reconstitution of human TPOR in murine cells restored STAT5 phosphorylation without any measurable changes in the dioxygenase activity. The presence or absence of the activated JAK/STAT signaling had no bearing on the TET-inhibitory effects of Epag. TPOR-independent TET-inhibitory action of Epag was further supported by the fact that another small-molecule TPOR agonist (Apag) and rTPO activated STAT5 but had no effect on TET-dioxygenase activity measured in the genomic 5hmC content.

Consistent with its inhibitory effect on TET2 activity, Epag treatment significantly increased the number of CFUs in *Tet2^+/+^* murine HSPCs, whereas this effect was completely absent in *Tet2^–/–^* bone marrow cells. As expected, lower activity of *Tet2* was associated with a higher baseline expansion rate, and the effect of Epag was not observed in cells with low TET2 activity. This genotype-dependent effect of Epag was even more profound in vivo. Epag improved recovery of *Tet2^+/+^* murine bone marrow recipients in syngeneic transplant in CD45.1 Pep Boy mice, whereas no significant effect was observed in mice receiving *Tet2^–/–^* grafts, suggesting that the effect of Epag can be attributed to TET2 inhibition. In a competitive bone marrow reconstitution with a split *Tet2^+/+^* and *Tet2^–/–^* graft, Epag provided a proliferative advantage to *Tet2^+/+^*, leading to a significant decrease in *Tet2^–/–^* fractions of blood cells. Further analysis of different lineages reinforced the notion that Epag treatment mimics loss of *Tet2,* as evidenced by myeloid skewing involving monocyte and neutrophil populations and increased numbers of LSKs and GMPs in *Tet2^+/+^* fractions with a concomitant decrease in *Tet2^–/–^* fractions of these cells.

Consistent with the TET-inhibitory action of Epag, analysis of the genomic DNA of the PBMCs of patients with aplastic anemia demonstrated a treatment-associated increase in the global cytosine methylation, an effect comparable to that observed in normal bone marrow in vitro but not with the structurally unrelated TPO-R agonist, Apag or rTPO. This TET2-inhibitory effect of Epag significantly prolonged the clonogenic potential of TET2-proficient bone marrow cells compared with rTPO, particularly in the second and third plating, with a higher proportion of CD11b^+^/CD14^+^ cells reinforcing the notion that Epag may be mimicking loss of TET2 in hematopoietic cells (31). We counted all colonies in the dish of human Methocult H4435 from STEMCELL Technologies. The Methocult H4435 used in our assay was expected to support the growth of erythroid progenitor cells (BFU-E and CFU-E); GMP cells (CFU-GM, CFU-G, and CFU-M); and multipotential granulocyte, erythroid, macrophage, and megakaryocyte progenitor cells (CFU-GEMM) colony. The addition of Epag also supports the growth of megakaryocytic progenitors (14), which makes it very difficult to differentiate different colony types based on the visual characteristics, so we counted all types of colonies in the different conditions. Furthermore, to objectively determine the impact of experimental conditions, i.e., Epag treatment, we subjected the harvested colonies for characterization by flow cytometry for the cellular output, including granulocytic and monocytic cells. This goes beyond the standard of colony counting with all of its inherent subjectivity, including visual assessment. This in vitro observation was consistent with the in vivo effect of Epag treatment on C57BL/6J *Tet2* WT mice. Serial replating experiments demonstrated the self-renewal capacity of stem and progenitor cells. This assay has been widely used to demonstrate the effects of various manipulations of genetic machinery in hematopoiesis, especially for the study of TET2 biology. Previous studies showed that TET2 deletion does not significantly affect CFUs in the first plating but in later serial replating, demonstrating TET2’s function in the self-renewal of HSPCs (29, 36). Consistent with these observations, we found that Epag significantly increased CFUs in the second/third plating but not the first plating. Several TPO-R agonists demonstrate similar hematopoietic activities in aplastic anemia, including TPO peptide mimetic romiplostim (37, 38), which does not inhibit TET2. In the multilineage recovery of patients with aplastic anemia, the TET-inhibitory effect of Epag may be secondary to TPO-R activation. However, consistent with the durable, unique clinical response of Epag in aplastic anemia, TET2 inhibition may be a key mechanism of action that contributes to a prolonged expansion of HSPCs, leading to complete response and recovery.

TET dioxygenases are critical regulators of cytosine methylation and thereby a gatekeeper for efficient transcription in mammalian cells (39–41). Here, we observed that there was no proliferative advantage of Epag treatment in the absence of TET2, which is very different from its activity in TET2-proficient cells. Recent reports showed that Epag restricts the clonal outgrowth of malignant cells in general (17, 22, 42–44). Our observations with Epag are consistent with these reports and further indicate that the dioxygenase inhibitory effect of Epag may be contributing to its antineoplastic effect. This is further supported by evidence that TET2-deficient leukemia cells rely on residual TET activity coming from TET1 and TET3 for their proliferative advantage and survival (23, 32). Thus, a further inhibition of TET activity may preferentially restrict the growth of *TET2^MT^* malignant cells while giving growth advantage to TET2-proficient, normal HSPCs (23, 32, 39).

However, the effect may be more complicated because the on-target effect on TPOR may provide a prosurvival signal by JAK/STAT signaling activation in human cells. Interestingly, analysis of previously reported clinical trial data is in agreement with our hypothesis that TET2 mutant cell response to Epag-mediated TET inhibition is in part due to Epag’s growth-promoting effects on WT HSPCs and restrictive effect on TET2 mutant cells. Thus, Epag may provide a competitive advantage to normal HSPCs while preventing outgrowth of TET2 mutant malignant cells. Another way to investigate whether Epag restricts the growth of *TET2* mutant cells would be to use mouse models of *TET2^MT^* clonal hematopoiesis of indeterminate potential to determine whether the malignant evolution of TET2 clone can be restricted or slowed by Epag.

The inhibitory properties of Epag on TET2 as they relate to its efficacy in aplastic anemia may in part explain the increased risk of clonal evolution to MDS, characterized by a high prevalence of monosomy-7 in certain individuals (45). Previously, Epag in combination with 5-azacytidine (5-Aza) was found to increase the risk of progression in the setting of MDS, leading to inferiority when the combination was compared with 5-Aza alone (46). Somatic LOF *TET2* mutations are common precursor lesions in MDS and also occur in clonal hematopoiesis of indeterminate potential, an asymptomatic condition credited with increased risk of myeloid neoplasia. It was reported that LOF *Tet2^MT^* facilitates the acquisition of subsequent mutational hits (47). Interestingly, the clinical trial of Epag in MDS was in combination with 5-Aza, known to upregulate *TET2* and *TET3* (48, 49), and thus Epag may have counteracted the effect of 5-Aza. It is possible that the increased risk of clonal evolution that we observed may be related to the TET-inhibitory effects of Epag in TET2-proficient cases that phenocopy LOF *TET2^MT^*. The opposing effects of these 2 drugs may in part explain the relatively inferior outcome in patients receiving the combination compared with the 5-Aza placebo.

In summary, our results indicate that Epag is a clinically relevant DNA dioxygenase inhibitor and this activity may contribute, in addition to its TPOR agonistic effect, to its efficacy in aplastic anemia, including reexpansion of HSPCs. This activity is exclusive to Epag and not observed with thrombopoietin or other TPO-R agonists. As such, Epag represents a class of agents with pleiotropic activity as TET dioxygenase inhibitors, conferring broader effects that may prove useful in many applications.

## Methods

### Key reagents.

The following reagents were used in this study: Epag, MedKoo Biosciences, 100941; DFO, Sigma-Aldrich D9533; Apag, MedChemExpress, HY-13463; rTPO, PeproTech, 300-18; and recombinant murine TPO (rmTPO), PeproTech, 315-14.

### Mice maintenance and experiments.

Animal care and procedures were conducted in accordance with Cleveland Clinic institutional guidelines and approved by the IACUC, Cleveland Clinic. *Tet2* mutant mice (stock 023359) were procured from The Jackson Laboratory. In mouse transplant experiments, recipient WT CD45.1 Pep Boy mice received 2 doses of 480 rad (4.8 Gy) irradiation delivered 3 hours apart followed by tail vein injection of 2 million donor bone marrow cells for each mouse. In single transplantation, 100% CD45.2 WT or *Tet2^–/–^* bone marrow cells were used as donor cells. In competitive transplantation, 5% *Tet2^–/–^* CD45.2 cells from C57BL/6J mice and 95% *Tet2*^+/+^ from CD45.1 Pep Boy cells were used as donor cells. Treatment (50 mg/kg Epag freshly dissolved in water or vehicle p.o. once a day, 5 days/week) started 24 hours after transplant in single experiments and 2 weeks after transplant in competitive experiments. Peripheral blood was collected by retro-orbital bleeding for complete blood count by Hemavet 950 (Drew Scientific) and/or flow cytometry analysis by FACSVerse (BD Biosciences) every month. Mice were euthanized for bone marrow analysis after 3 months of treatment. Antibodies used in this study for flow cytometry analysis are presented in Supplemental Table 2.

### Tissue culture.

Human normal bone marrow samples and cord blood cells were collected from healthy donors in accordance with Cleveland Clinic IRB-approved protocols. Mononuclear cells were purified by Ficoll (Histopaque-1077, Sigma-Aldrich, 10771) from bone marrow and cord blood samples. CD34^+^ cell purification was performed using CD34 MicroBead Kit (Miltenyi Biotec, 130-097-047). All human primary mononuclear cells, including CD34^+^ cells, were maintained in IMDM supplied with 20% FBS, 100 U/mL penicillin-streptomycin, and 100 ng/mL recombinant human SCF (PeproTech, 300-07).

Mouse bone marrow cells after red blood lysis were maintained in IMDM supplied with 20% FBS, 100 U/mL penicillin-streptomycin, and 50 ng/mL recombinant murine SCF (PeproTech, 250-03). 32D and Ba/F3 cell lines were purchased from ATCC and maintained in IMDM culture medium supplied with 10% FBS, 100 U/mL penicillin-streptomycin, and 2 ng/mL murine IL-3 (PeproTech, 213-13).

32D and Ba/F3 cells were electroporated by Amaxa Nucleofector II with programs E-032 and X-001, respectively, with human TPO-R plasmid MPL-pCMV-3Tag-3a. The open reading frame of MPL (NM_005373.2) was cloned in pCMV-3Tag-3a vector to generate MPL-pCMV-3Tag-3a (GenScript). Stable cell lines were established in culture for 2 weeks in the presence of 500 μg/mL G418 (Gibco, 10131-035). TET2 mutant cells (T30 and T31), as well as their vector control cells (Vec), were generated and described earlier (23).

### Colony-forming assay.

Methocult M3434 (murine) and H4435 (human) from STEMCELL Technologies were used for colony-forming assays. Methylcellulose was supplied with the indicated concentration of Epag or rTPO before cell seeding. Mouse bone marrow cells after red blood lysis were seeded at the concentration of 30,000 cells/mL. Bone marrow mononuclear cells from healthy donors and patients were seeded at the concentration of 100,000 cells/mL. CD34^+^ cells were infected with lentiviral TET2 targeting shRNA or nontargeting *scr* shRNA as described and characterized in our previous study (31). Two days after lentivirus infection, CD34^+^ cells were seeded at the concentration of 5000 cells/mL in Methocult in the presence of 5 μg/mL puromycin. Colonies were scored and cells were harvested for replating or flow cytometry analysis by FACSVerse (BD Biosciences) on days 10 to 14.

### Expression and purification of recombinant catalytic domains of TET1, 2, and 3.

TET2 catalytic domain was purified in the lab. GST-TET2 (1099-1936 Del-insert; ref. 24) expression vector was transformed into *E*. *coli* strain BL21(DE3)pLysS. The transformant was grown at 37°C to OD 600 of 0.6 and switched to 16°C for 2 additional hours. Ethanol was added to the final concentration of 3% before induction by adding isopropyl-b-D-thiogalactopyranoside to the final concentration of 0.05 mM. Cells were cultured for 16 hours at 16°C. Cells from 2 L culture were harvested and lysed in 50 mL of lysis buffer (20 mM Tris-HCl pH7.6, 150 mM NaCl, 1× CelLytic B [Sigma-Aldrich, C8740], 0.2 mg/mL lysozyme, 50 U/mL Benzonase, 2 mM MgCl_2_, 1 mM DTT, and 1× protease inhibitor [Thermo Fisher Scientific A32965]) for 30 minutes on ice. Lysate was sonicated by an ultrasonic processor (Thermo Fisher Scientific FB-505 with ½” probe) with an amplitude of 70% for 18 1-minute cycles (20 seconds on and then 40 seconds off). The lysate was then centrifuged twice at 40,000*g* for 20 minutes. The supernatant was filtered through the membrane with a pore size of 0.45 μm. Flow through was diluted 4 times with the solution of 20 mM Tris-HCl pH7.6, 150 mM NaCl. GST-TET2 was purified by GE Healthcare AKTA Pure by affinity (GSTPrep FF16/10) and gel filtration (Superdex 200 Increase 10/300 GL). For gel filtration, buffer of 10 mM phosphate and 140 mM NaCl, pH 7.4, was used. Recombinant TET1 (Epigentek, E12002-1) and TET3 (BPS Bioscience, 50163) proteins were purchased and used without any further purification.

### 5hmC ELISA.

The 96-well microtiter plate was coated with 10 pmol avidin (Sigma-Aldrich, A8706) in 0.1 M NaHCO_3_ at a pH of 9.6 in 100 μL followed by biotin-5mC-DNA (IDT) substrate capture at room temperature. TET2^CD^ protein (0.4 μg) in 100 μL assay buffer (50 mM HEPES pH 6.5, 100 mM NaCl, 0.1 mM Fe(NH4)_2_(SO4)_2_ or FeCl_3_, and varying concentrations of the indicated drugs along with 1 mM 2-OG) was added to each well for 2 hours at 37°C. Reactions were stopped using 0.05 M NaOH (100 μL) on a shaking platform for 1.5 hours at room temperature. After washing, wells were blocked with 2% BSA in TBST for 30 minutes and incubated with anti-5hmC antibody (active motif, 39769, 1: 3000) at 4°C overnight. After 4× washes, wells were incubated with HRP-conjugated anti-rabbit secondary antibody (Santa Cruz Biotechnology) and developed by adding 3,3′,5,5′-Tetramethylbenzidine Liquid Substrate (Sigma-Aldrich, T4444). Reactions were stopped by adding 2M H_2_SO_4_. ODs were recorded at 450 nm. TET2 inhibitor screening was performed in 384-well plates.

### SPR.

Kinetic characterization of TET2 binding to Epag was monitored by SPR with a Biacore 3000 (GE Healthcare). Response units (RUs), a measure of binding, were monitored as a function of time. To prepare a surface plasmon sensor chip, purified GST-tagged TET2^CD^ (purity >90%) was captured by anti-GST antibodies as described previously (31, 50). Varying concentrations of Epag (0–100 μM) in the presence of 25 μM Fe^2+^ or Fe^3+^ and 25 μM 2-oxoglutarate were used as analytes. In all SPR experiments, analyte solutions of different concentrations were passed over the sensor chip containing immobilized protein at a flow rate of 10 μL/minute for 5 minutes, and dissociation was monitored while SPR buffer passed over the chips for an additional 5 minutes. Data were normalized against a reference channel containing immobilized GST. Surfaces were regenerated using 1 injection of 1 M NaCl in 10 mM NaOH at 40 μL/minute for 30 seconds. Analysis and fitting of data were performed with BIAevaluation software, version 3.2 (Biacore Inc.), with the option for simultaneous Ka/Kd calculations. Sensorgram data were fitted using global fits to yield Ka and Kd simultaneously assuming a 1:1 Langmuir model. Goodness of fit was acceptable based on the criterion of χ^2^ ≤ 1% of the observed maximum response (R_max_).

### Computational docking and in silico structural analysis.

The crystal structure of TET2^CD^ in complex with 5mCpG containing DNA oligo, N-oxalylglycine (NOG), and Fe^2+^ (Protein Data Bank ID 4NM6) was used to dock 3-dimensional–optimized Epag. In silico docking experiments of TET2 and aplastic anemia were performed using Glide in the computational environment Maestro running on a Quantum TXR411-0128R graphical processing unit. Initially, the grid sizes were kept large enough to contain the entire molecule. The most favored binding poses of aplastic anemia with TET2 were determined by restricting the NOG/Fe^2+^ binding site. The complex was minimized, and the binding poses were analyzed in UCSF Chimera 1.8.

### Spectrophotometric quantification of Epag.

For cell suspension, cytoplasmic extract, and nucleus suspension preparation, cells of 32D were treated with indicated concentrations of Epag or left untreated in plain RPMI culture media at a density of 1 million/mL for half an hour. Then, cells were harvested after treatment and suspended in buffer A (10 mM HEPES, pH 7.8, 10 mM KCl, 1.5 mM MgCl_2_, 0.34 M sucrose, 10% glycerol) at concentration of 2.5 million/mL (cell suspension). NP40 at final concentrations of 0.2% was added to cells, and cells were vortexed for 10 seconds at the highest setting (Vortex Genie-2, Scientific Instruments). Supernatant was removed to a new tube (cytoplasmic extract) after centrifugation (5 minutes, 1300*g*, 4°C). Nuclei pellet was washed twice with buffer A and suspended at the same volume of buffer A of cytoplasmic extract (nucleus suspension). To obtain the UV-visible spectroscopic property of Epag, gradient concentrations of Epag were made in different solutions and subcellular fractions prepared from untreated 32D cells. Solution of 100 μL was added to a UV transparent plate (Corning, 3635) and absorbance from 300 nm to 700 nm with 1 nm per step was measured Synergy H1 Hybrid Reader (BioTek).

### Protein extraction and Western blot.

Cell pellets were lysed in RIPA buffer (Thermo Fisher Scientific, 89900) supplied with 1× protease inhibitor cocktail (Thermo Fisher Scientific, A32965), 5 mM EDTA, and 1× phosphatase inhibitor cocktail 2&3 (Sigma-Aldrich, P0044 & P5726) on ice for 15 minutes. Lysate was sonicated by an ultrasonic processor (Thermo Fisher Scientific FB-505 with 1/8” diameter probe) with a setting of 3 for 5 cycles (5 seconds on and then 5 seconds off). After centrifuging (15 minutes, 20,000*g*, 4°C) supernatant was collected for Western blot analyses. STAT5 (Cell Signaling Technology, 94205T), p-STAT5 (Cell Signaling Technology, 9351S), Flag (Sigma-Aldrich, F1804), β-actin (Cell Signaling Technology, 4967S), GAPDH (Cell Signaling Technology, 8884S), and Histone H3 (Upstate, 06-755) antibodies were used.

### Dot blot.

Genomic DNA was extracted using the Promega purification kit (A1620). DNA samples were denatured in denaturing buffer (0.4 M NaOH/10 mM EDTA) for 10 minutes at 95°C and neutralized with equal volumes of 2 M NH4OAc (pH 7.0). The DNA was then spotted on a nitrocellulose membrane using a Bio-Dot Apparatus Assembly (Bio-Rad). The membrane was air dried, cross-linked by Spectrolinker XL-1000 (120 mJ/cm^2^), and detected with anti-5hmC (active motif, 1:5000) or anti-5mC (Eurogentec, 1:2500) antibodies. The membrane was stained with methylene blue.

### Statistics.

All statistical analyses were performed in GraphPad Prism 8.0 unless otherwise described. Statistical significance testing was performed using 2-tailed Student’s *t* test unless described otherwise. To compare multiple experiments, 1-way ANOVA with Dunnett’s test was performed. For each case, a *P* value less than or equal to 0.05 was considered significant. Each experiment was performed in triplicate at least twice wherever possible.

### Study approval.

Animal care and procedures were conducted in accordance with institutional guidelines and with a protocol approved by the IACUC of Cleveland Clinic. Human patient samples used in this study were collected for research purposes using written informed consent in accordance with Cleveland Clinic IRB-approved protocol.

### Data and material availability.

All requests for raw data and specific materials, including engineered stable cell lines reported in this manuscript, can be made to the corresponding authors. Results of individual repeats are provided as Supplemental Figure 4.

## Author contributions

YG designed research studies, performed experiments, acquired and analyzed data, and wrote parts of the manuscript. MH, DJ, BP, SP, DRG, ADT, and SK collected data and provided reagents for the study. SS and TL performed data analysis. DL and YP helped with mouse experiments. MAS, YS, and OYM provided reagents and assisted in writing the manuscript. JPM conceived and conceptualized the study, read and edited the manuscript, and generated resources. BKJ conceived, conceptualized, and designed the study, supervised the research, acquired and analyzed data, developed the reagents, wrote the original draft, edited the manuscript, and generated resources for the study.

## Supplementary Material

Supplemental data

Supplemental table 1

Supplemental table 2

## Version 1 01/27/2022

In-Press Preview

## Version 2 02/15/2022

Electronic publication
