# Supplementary material for: Eltrombopag inhibits TET dioxygenase to contribute to hematopoietic stem cell expansion in aplastic anemia
Source: J Clin Invest. 2022 Feb 15;132(4):e149856. doi: 10.1172/JCI149856 (PMC8843742; doi:10.1172/JCI149856)
Supplement: Supplemental data [file jci-132-149856-s103.pdf]

Supplemental Figure 1

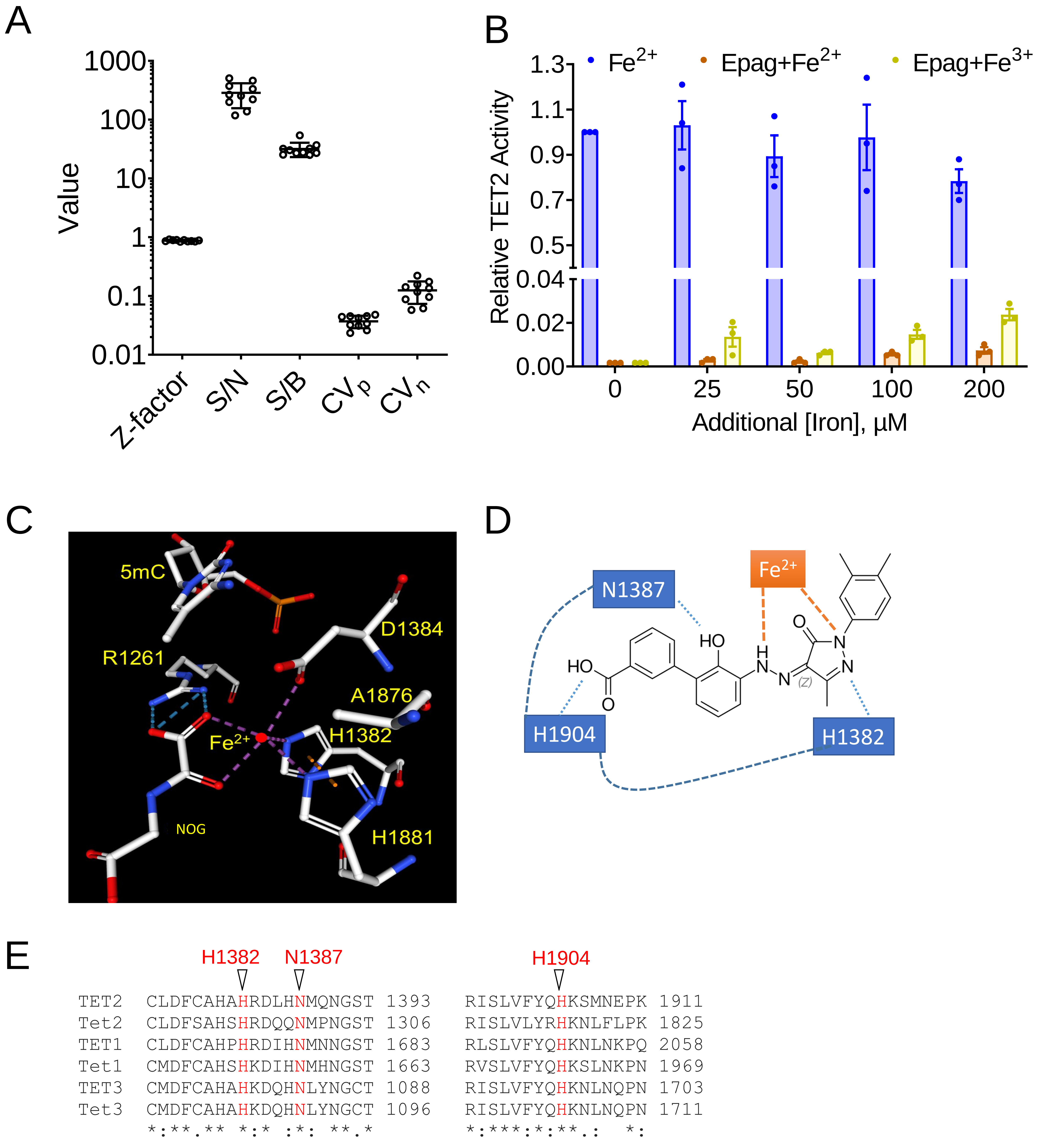

Supplemental Figure 1. Screening and validation of Epag as a TET-dioxygenase inhibitor. (A) Assay optimization and reproducibility and stability. The plot showing the Z-factor defined as 1-3(δp+δn)/ |µp - µn|, where µ is means, δ is standard deviations of both the positive (p) and negative (n) controls; the signal-to-noise ratio S/N=|µp - µn|/ δn; signal-to-background ratio S/B =µp/µn and coefficient of variation CV =δ/µ. (B) Addition of either form of iron could not rescue the TET enzymatic activity. TET2 ELISA was performed with 25 µM Epag and 25 µM Fe2+. The addition of either Fe2+ or Fe2+ in the assay was indicated in Figure. (C) Iron coordination in TET2 active site in the absences of Epag. (D) Diagram shows the interaction Epag, Fe, and TET2 based on in silico docking. (E) CLUSTAL O (1.2.4) multiple sequence alignment (http://www.ebi.ac.uk/). Histidine (H) 1382, asparagine (N) 1387, and histidine (H) 1904. Following sequence from NCBI was used for the alignment: Homo sapiens TET1 (NP_085128.2), TET2 (NP_001120680.1) and TET3 (NP_001274420.1); Mus musculus Tet1 (NP_001240786.1), Tet2 (NP_001035490.2) and Tet3 (NP_001334242.1). (B) Results are representative of three independent experiments performed and are expressed as mean ± SEM of three replicates.

Supplemental Figure 2

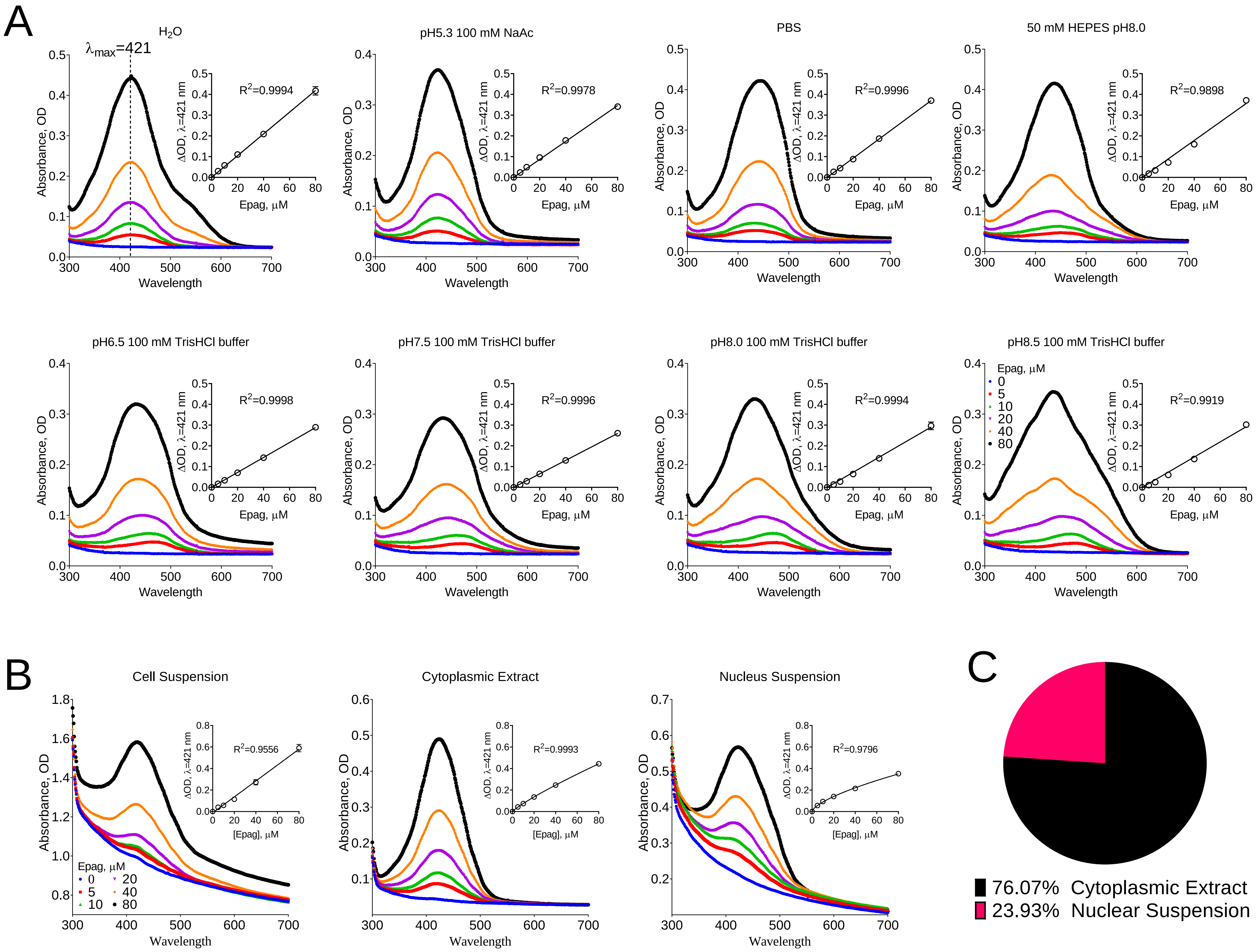

Supplemental Figure 2. Eltrombopag in different subcellular fraction. (A-B) Absorption maxima and standard curves of Epag absorbance at 421 nm in different conditions. The absorption spectrum was measured in uv-visible spectrophotometer Synergy H1 Hybrid Reader (BioTek, step: 1nm, speed: sweep). (C) Distribution of Epag into different subcellular compartments. The concentration fractions were calculated by dose-response nonlinear regression presented in (B) and plotted in Pi-chart. (A-B) Results are representative of three independent experiments performed.

Supplemental Figure 3

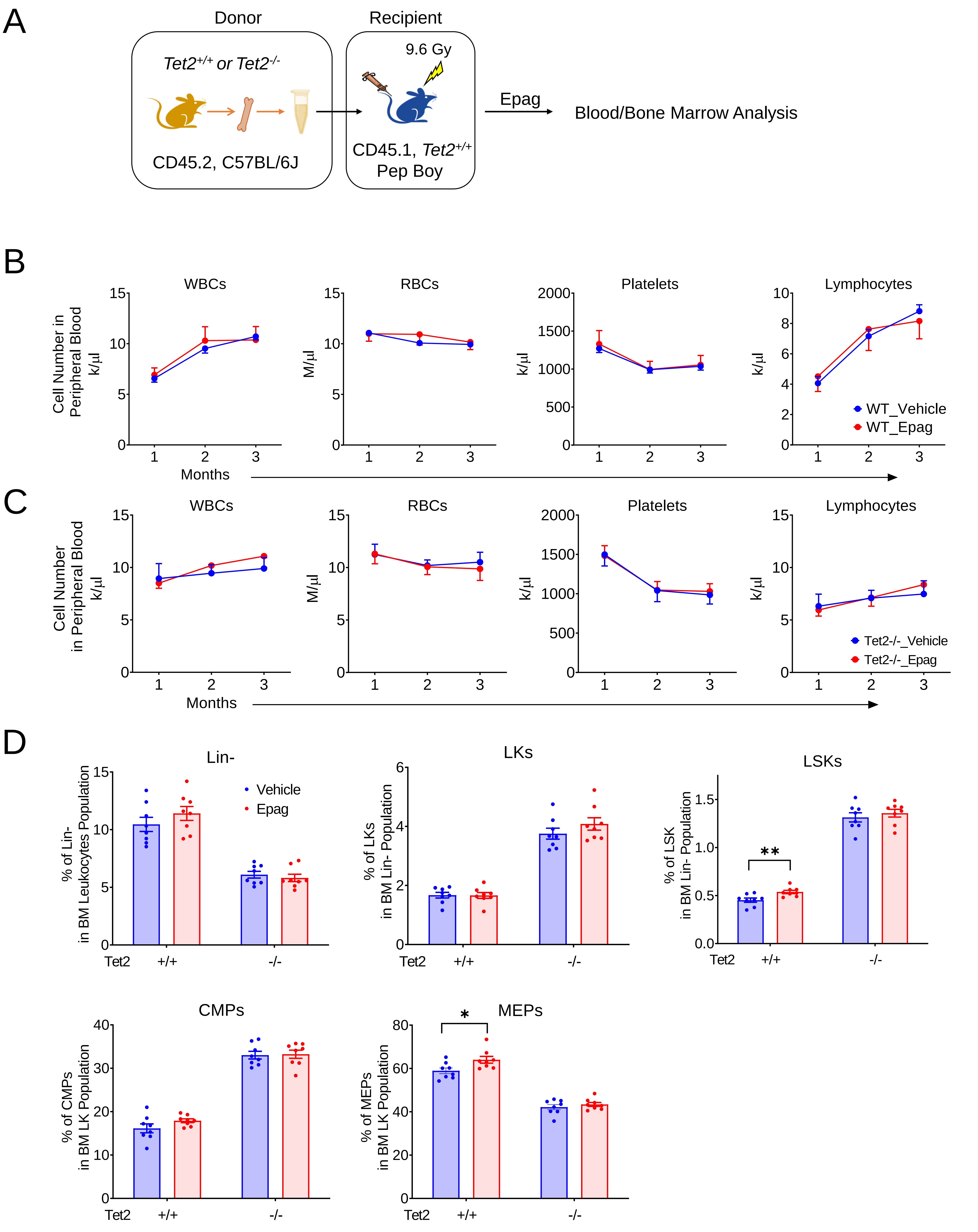

Supplemental Figure 3

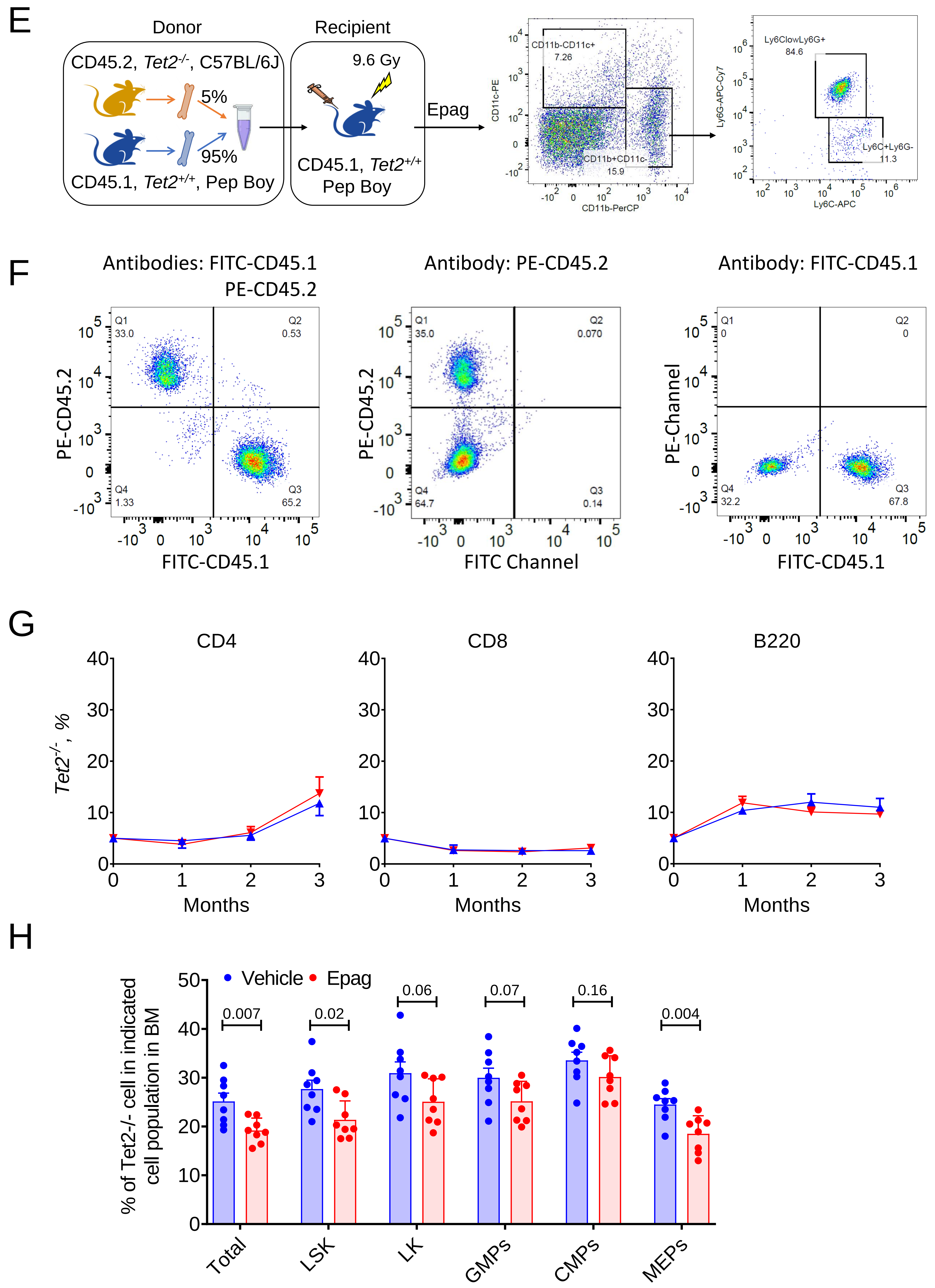

Supplemental Figure 3. Epag inhibits TET-dioxygenase and mimics loss of TET2 (**A**) Murine bone marrow derived mononuclear cells were treated with 10 µM (Epag 10) and 20µM (epag 20) eltrombopag and the colony forming assays were performed using Methocult™ (M3434, StemCell™ Technologies) known to support the growth of myeloid and erythroid colonies. Different colonies (BFU-E, CFU-G, CFU-M, CFU-GM and CFU-GEMM were counted in first and second plating, based on their morphologies as per the manufacturer guideline. The data is combination of two independent experiments performed using bone marrow derived mononuclear cells from 2 mice per group/treatment, p values are indicated compared to vehicle (DMSO, 0.1% V/V, control). Schema of experimental design of *Tet2*$^{+/+}$ or *Tet2*$^{-/-}$ transplant experiments for the data presented in (**B-D**). *Tet2*$^{+/+}$ CD45.1, Pep Boy mice were lethally irradiated and received 2 million of C57BL/6J *Tet2*$^{+/+}$ or *Tet2*$^{-/-}$ mice bone marrow cells through intravenous injection via tail veils. Mice were divided into two groups and either treated with 50 mg/kg Epag or vehicle (water) by oral gavage. Peripheral blood and bone marrow samples were collected for analysis. (**B** and **C**) Peripheral blood samples were counted by HemaVet (Drew Scientific). (**D**) Flow analysis of Lin^{-}, LSKs, LKs, CMPs, and MEPs populations in bone marrows. (**E**) Scheme of experimental design of *Tet2*$^{-/-}$ competitive repopulation transplantation experiments. PEP (CD45.1) mice were lethally irradiated and received 2 million bone marrow cells consist of 95% *Tet2*$^{+/+}$ (PEP, CD45.1) and 5% *Tet2*$^{-/-}$ (CD45.2) through intravenous injection via tail veils. Mice were divided into two groups and either treated with 50 mg/kg Epag or vehicle (water) by oral gavage after two weeks of transplantation. Flow cytometry pictures shows the gating strategy for CD11b^{+}CD11c^{-}Ly6C^{+}Ly6G^{-} (Monocytes) and CD11b^{+}CD11c^{-}Ly6C^{low}Ly6G^{+} (Neutrophils). (**F**) Single or double antibodies staining for CD45.1 and CD45.2 cells gave identical results. (**G**) Blood at different time points was harvested for flow cytometry analysis for CD4^{+}, CD8^{+} and B220^{+} cell populations. (**H**) Bone marrow analysis of different cell populations. (**E** and **F**) Results are representative of at least three independent experiments performed. (**B**-**D** and **G**-**H**) A total of 4 donor mice and 8 recipient mice were used per group/treatment in 2 independent experiments. Data are expressed as mean ± SEM of eight replicates. **P*<0.05 and indicated *P* values by 2-tailed unpaired t test.

Supplemental Figure 4

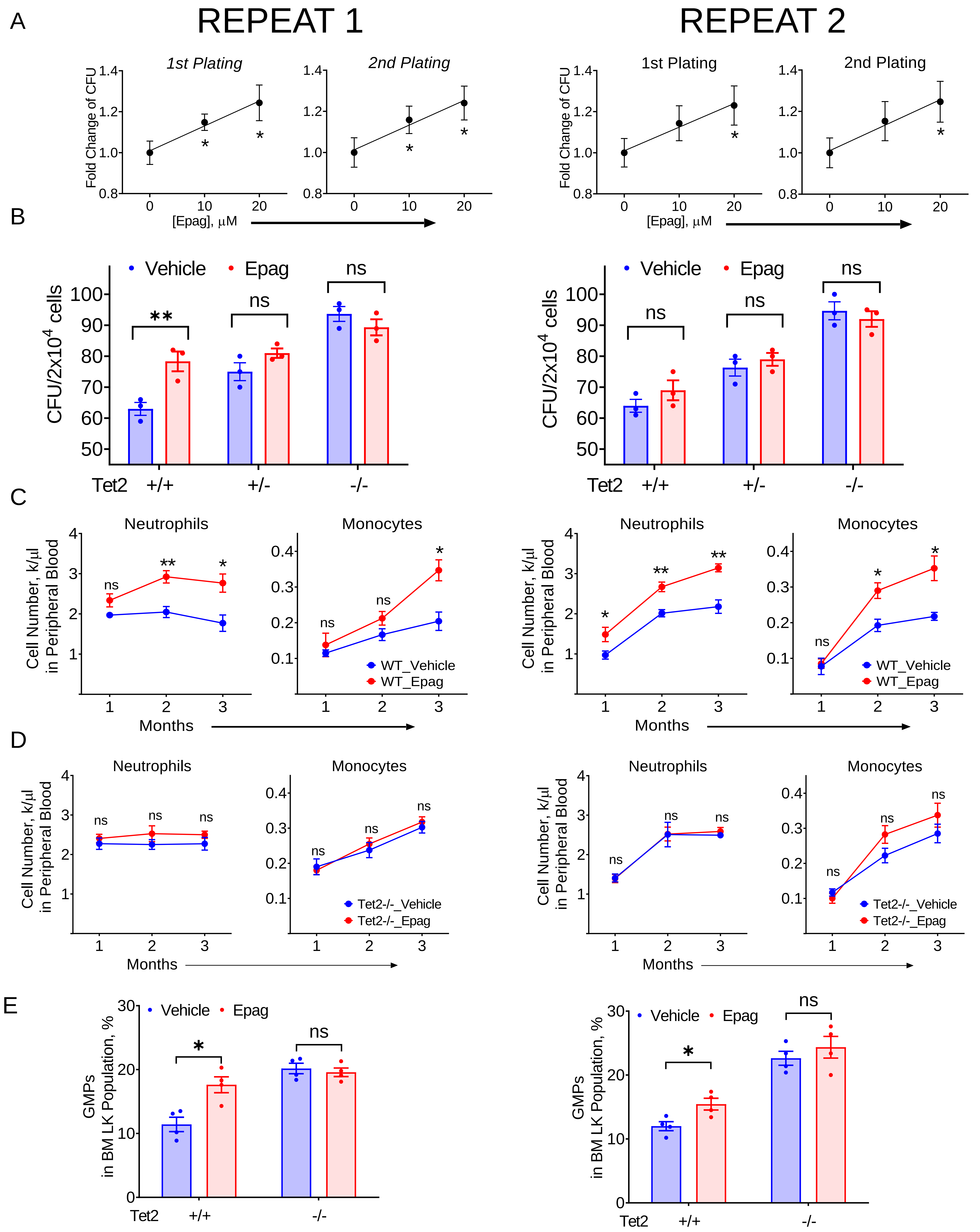

Supplementary Figure 4. A-D: Individual repeats experiments of the data presented in Figure 3 A-D and E presents the repeat data of Figure 3F.

Supplemental Figure 4

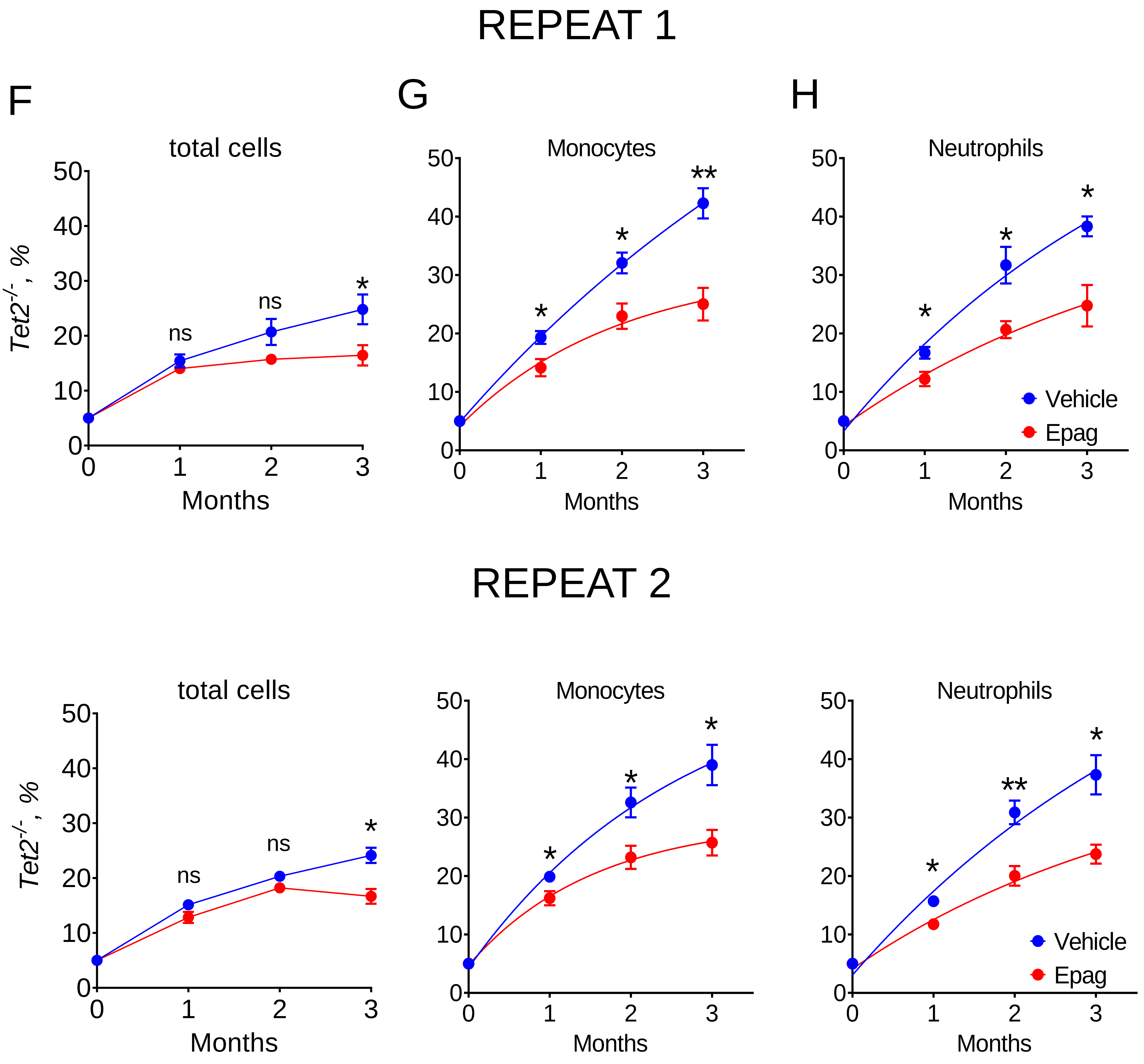

Supplemental Figure 4: F-H Individual repeat experimental data presented in Figure 3G-I

Supplemental Figure 4

I

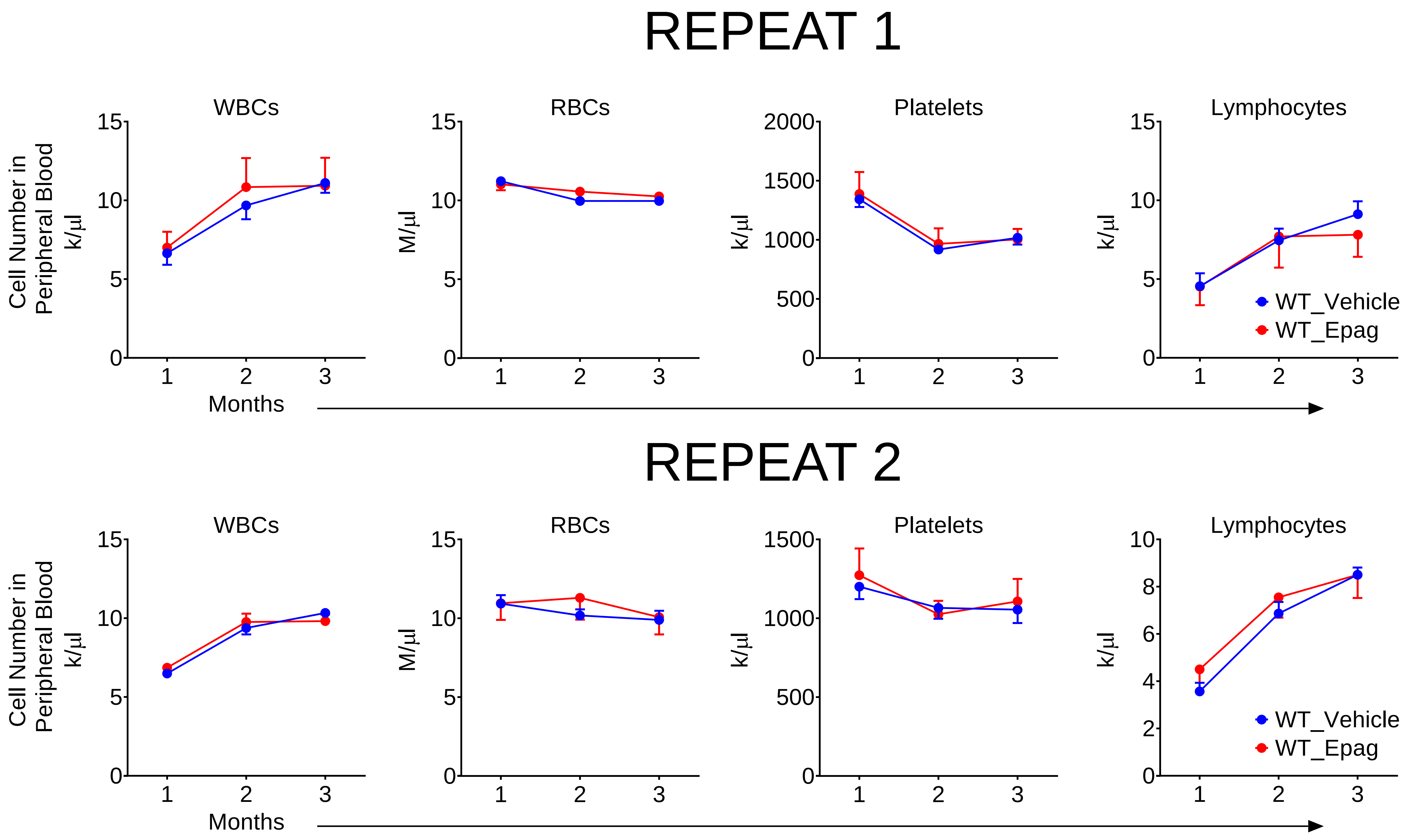

J

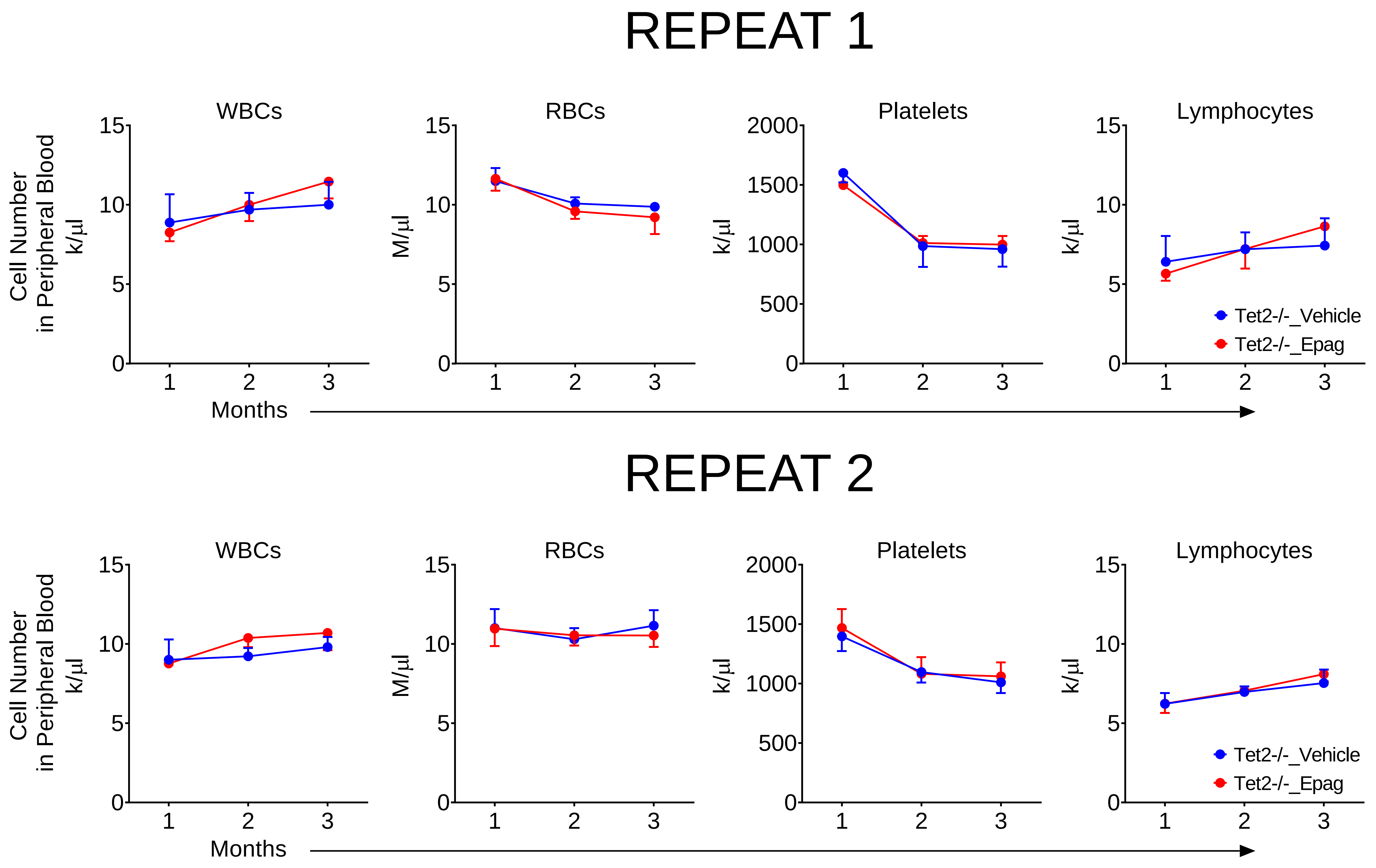

Figure 4 I-J Data of individual repeat experiments presented in supplemental figure S3B and S3C

Supplemental Figure 4

REPEAT 1

K

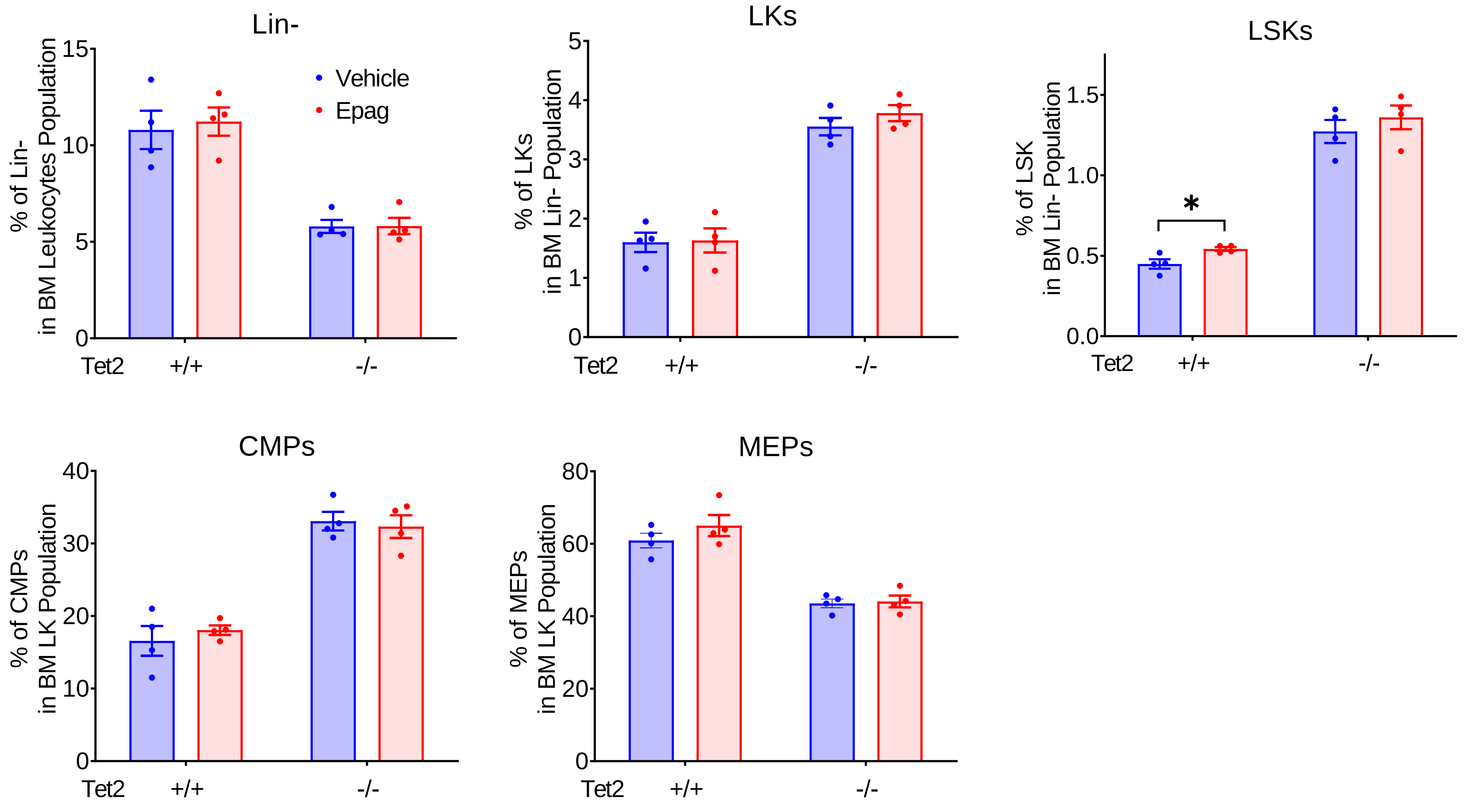

REPEAT 2

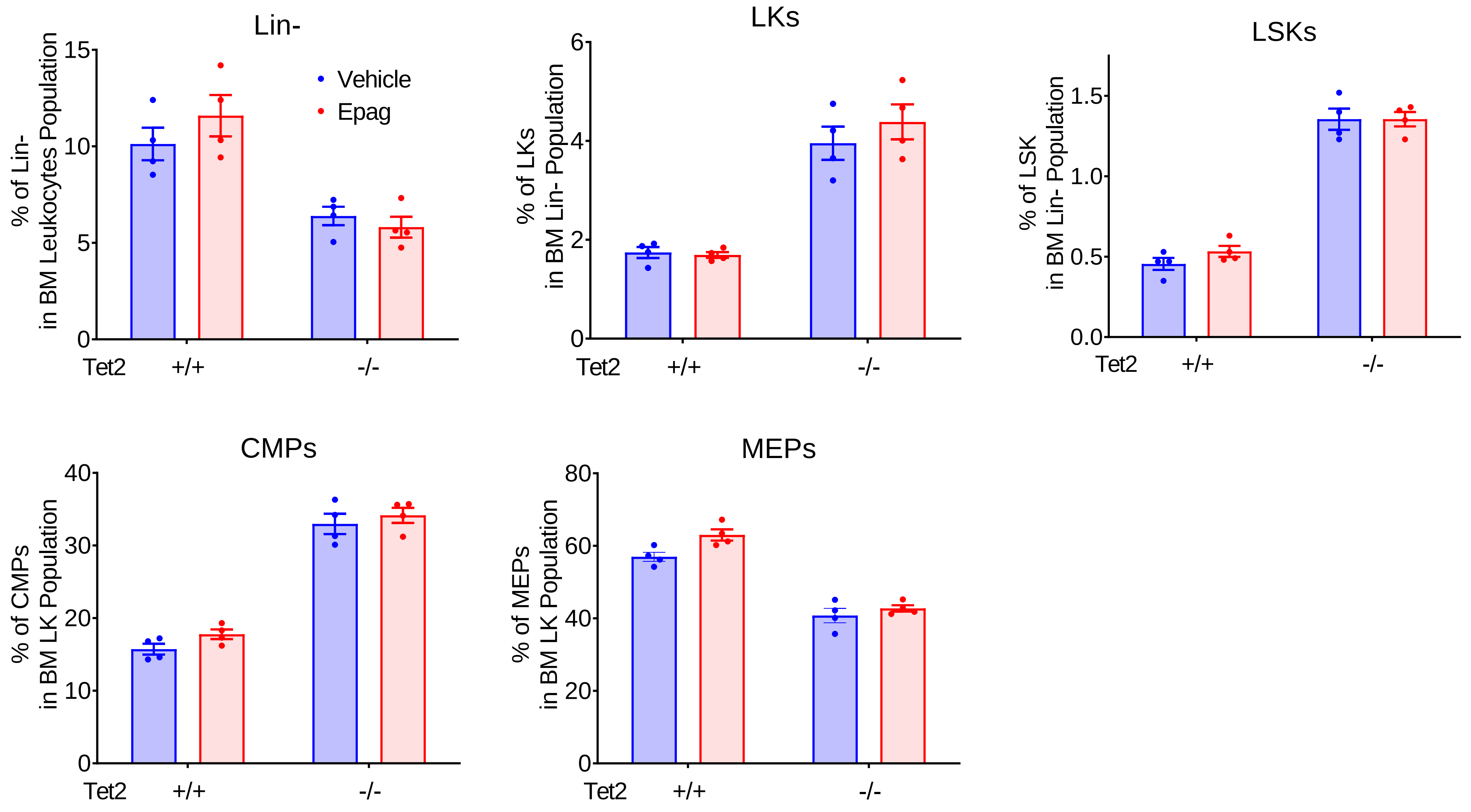

Supplemental Figure 4K: Data of individual repeat experiments presented in supplemental figure S3D

Supplemental Figure 4

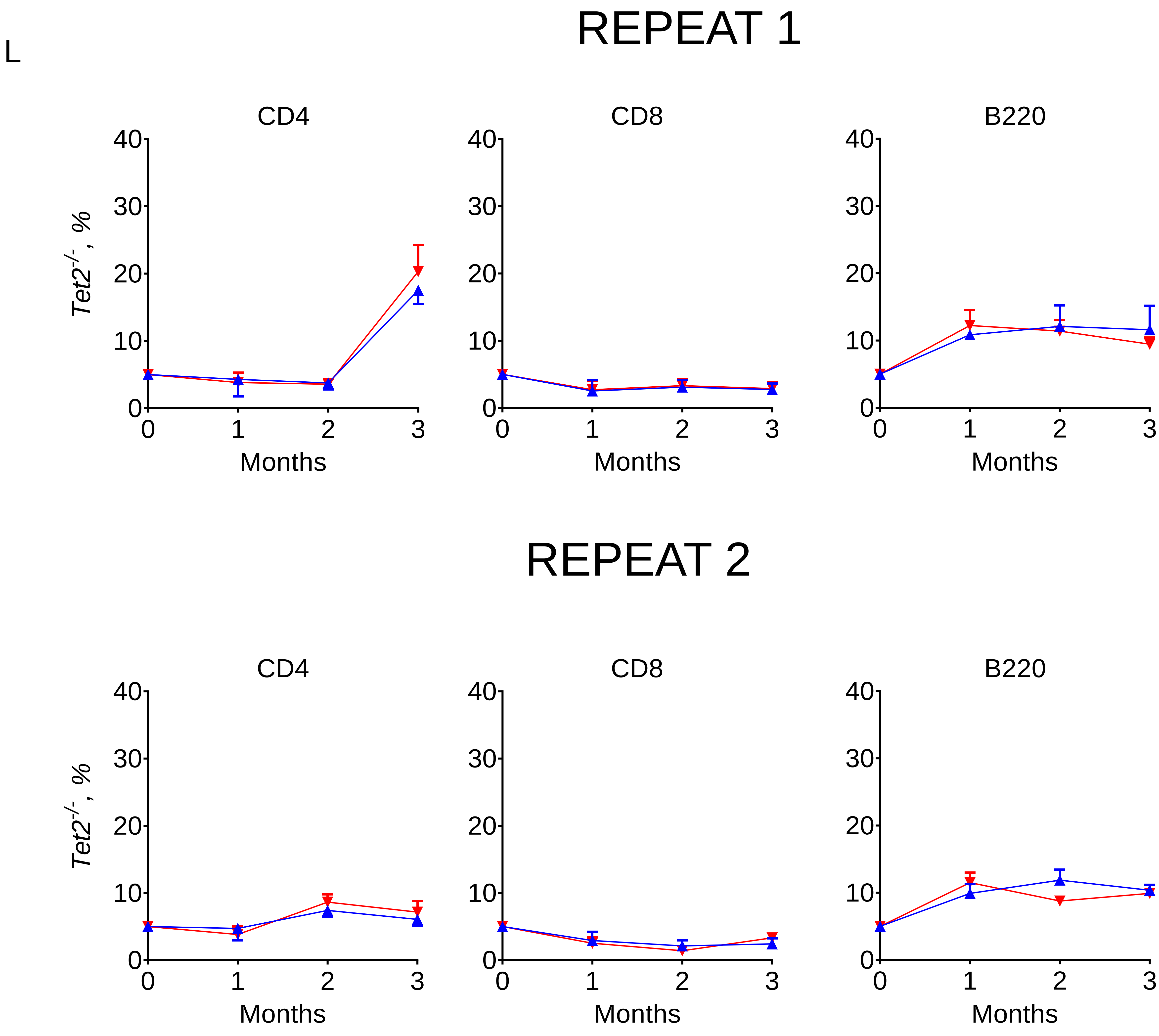

Supplemental Figure 4L: Data of individual repeat experiments presented in supplemental figure S3G

Supplemental Figure 4

M

REPEAT 1

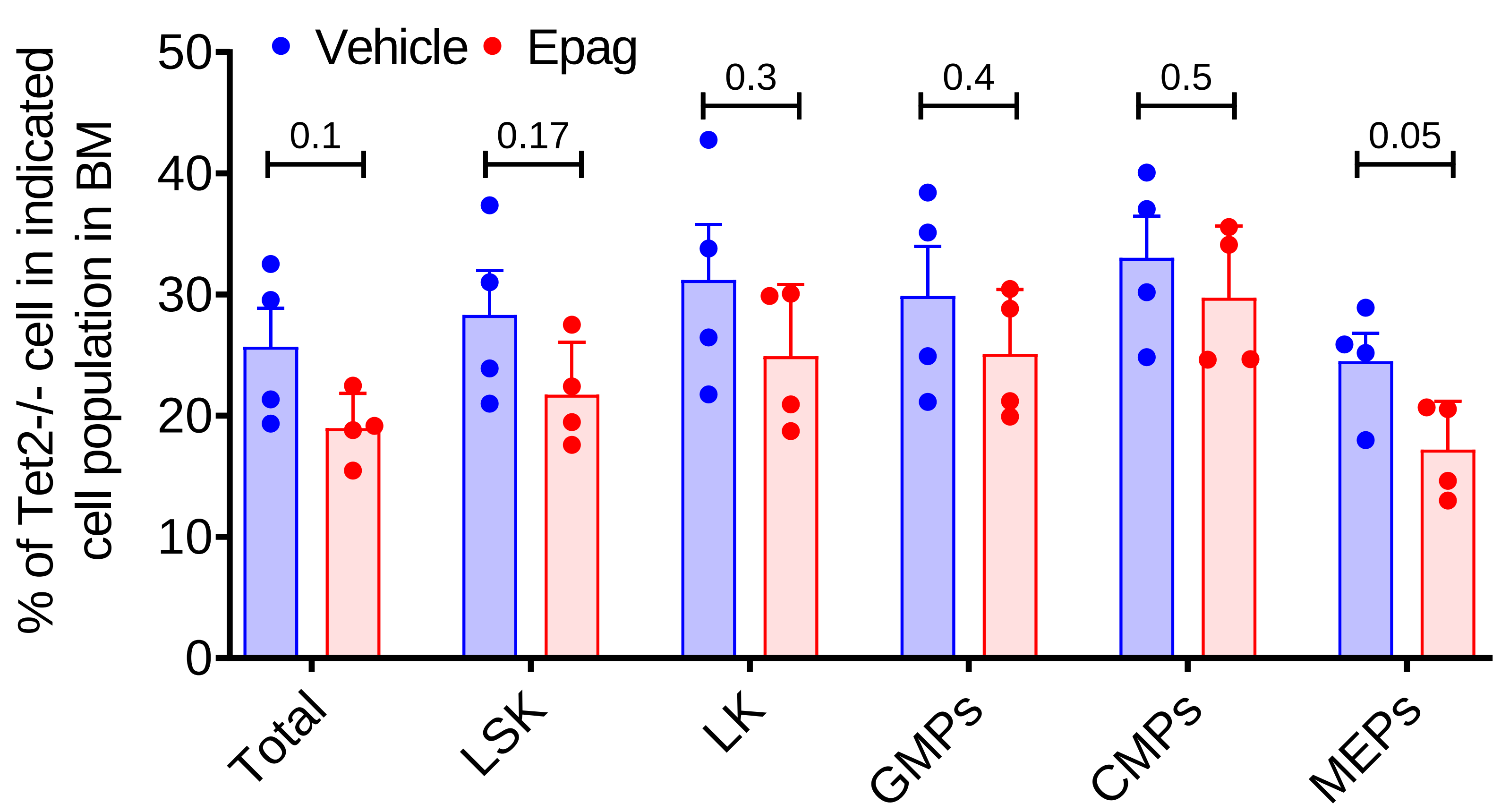

REPEAT 2

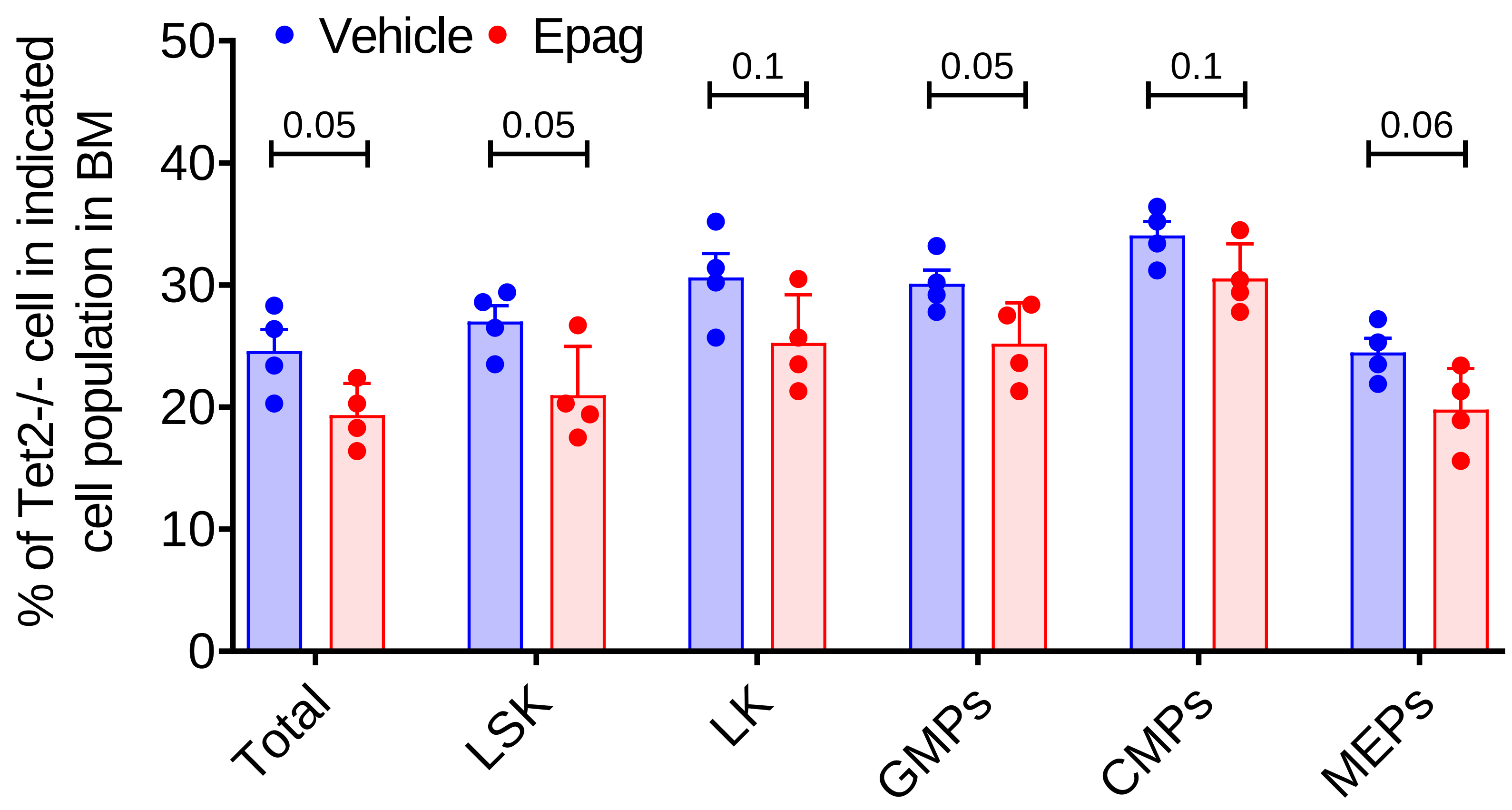

Supplemental Figure 4M: Data of individual repeat experiments presented in supplemental figure S3H

Supplemental Figure 5

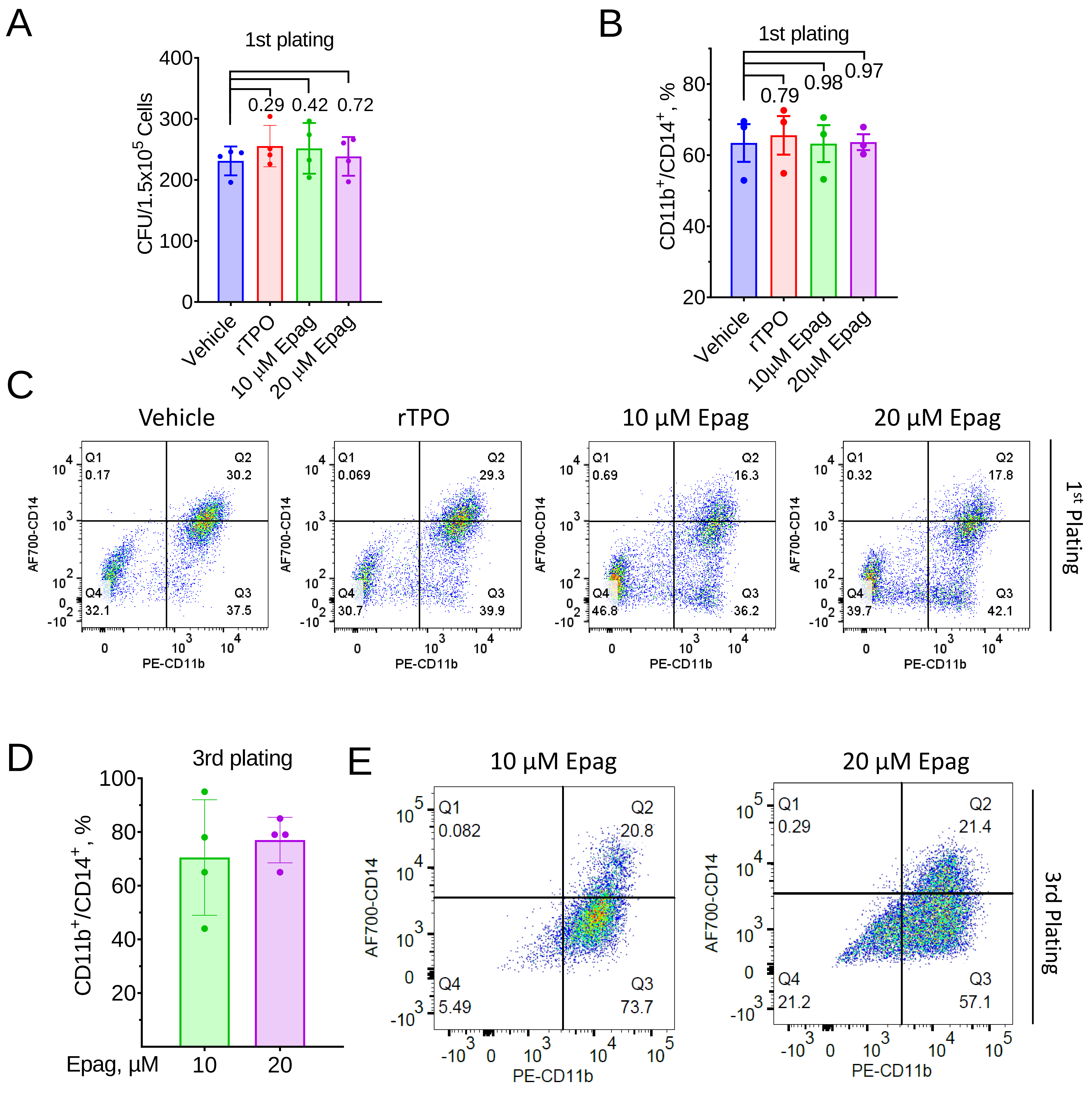

Supplementary Figure 5. Epag expands TET2 WT HSPCs (**A**) Colony-forming assay was performed for human bone marrow mononuclear cells from healthy donors (NBMs), n=4. Mononuclear cells were seeded in Methocult™ with indicated concentrations of Epag or 100 ng/ml rTPO. Colony-forming units were counted after the 1st plating. (**B**-**D**) Follow analysis of cells harvested from the 1st and 3rd plating of colony-forming assay in (**A**) and **Figure 4E**. (**C** and **E**) Results are representative of at least three independent experiments performed. Data are expressed as mean ± SEM of three (**B**) or four (**A** and **D**) biological replicates in 3 independent experiments. *P* values by 2-tailed unpaired t test are indicated.

Supplemental Figure 6

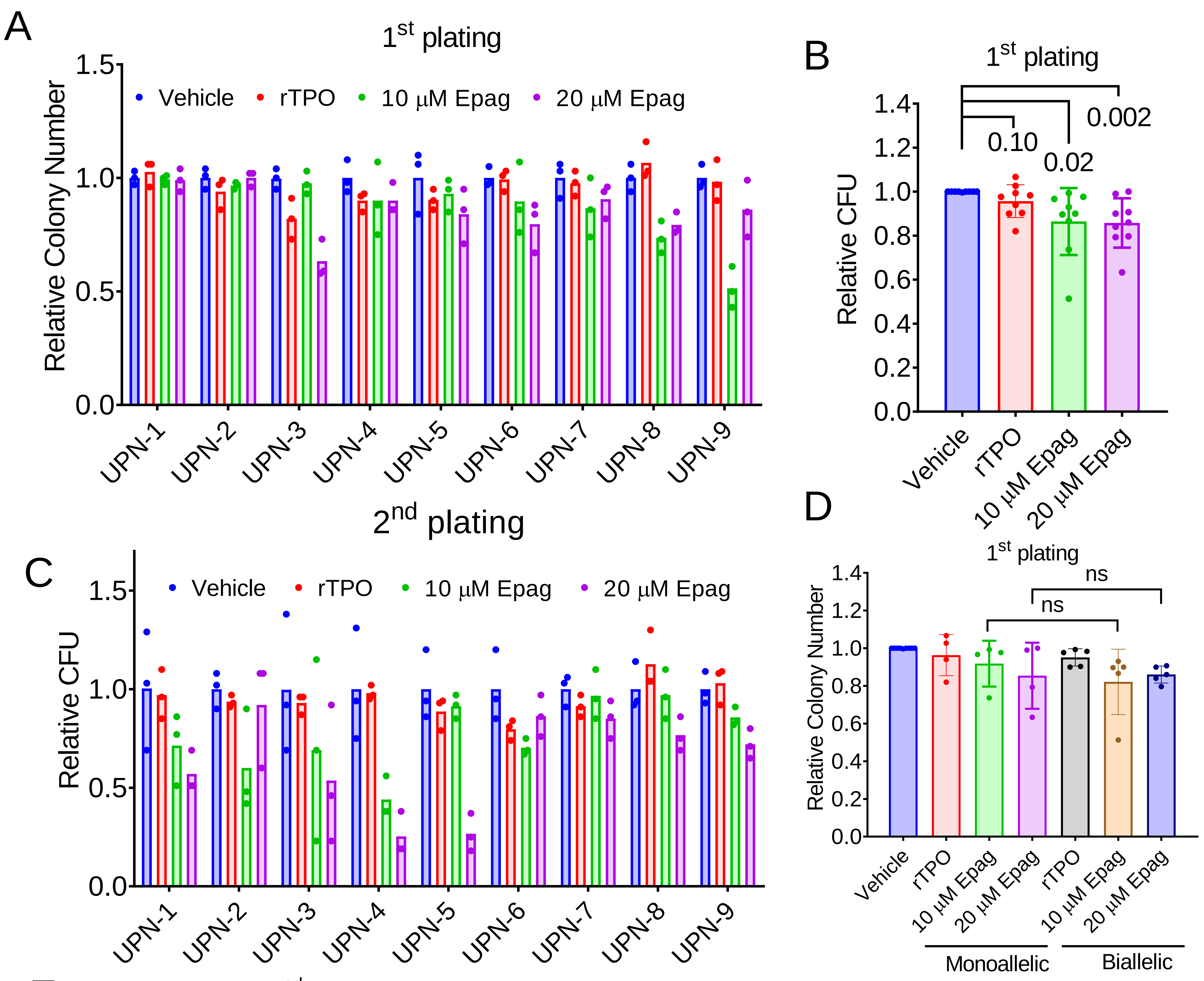

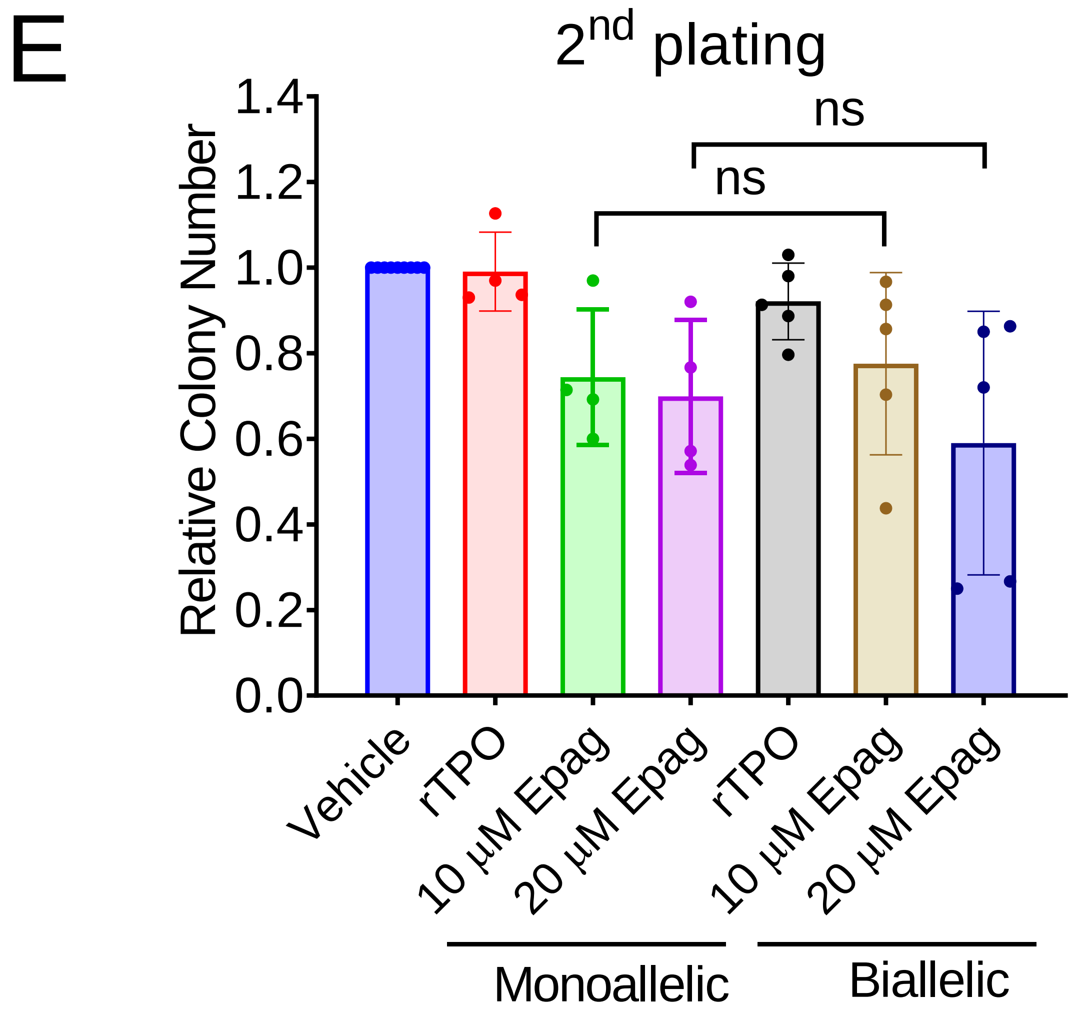

Supplementary Figure 6. Epag prevents clonal outgrowth of *TET2^{MT}* primary bone marrow derived mononuclear cells from MN patients. Mononuclear cells were purified from myeloid neoplasm patient bone marrows (n=9) with TET2 mutations. Mononuclear cells were seeded in Methocult™ with indicated concentrations of Epag or 100 ng/ml rTPO. Colony-forming units were counted after 1st (**A**-**B**) and 2nd (**C**) plating, and relative colony numbers were plotted. (**D**-**E**) Relative colony numbers from 1st and 2nd platings were plotted based on TET2 mutation status, monoallelic or biallelic. (**A** and **C**) Data of three technical replicates and their means are shown. Data are expressed as mean of nine biological replicates in three independent experiments for (**B**). *P* values and ns (*P*>0.05) by 2-tailed unpaired t test are indicated.

Full unedited blot for Figure 2B

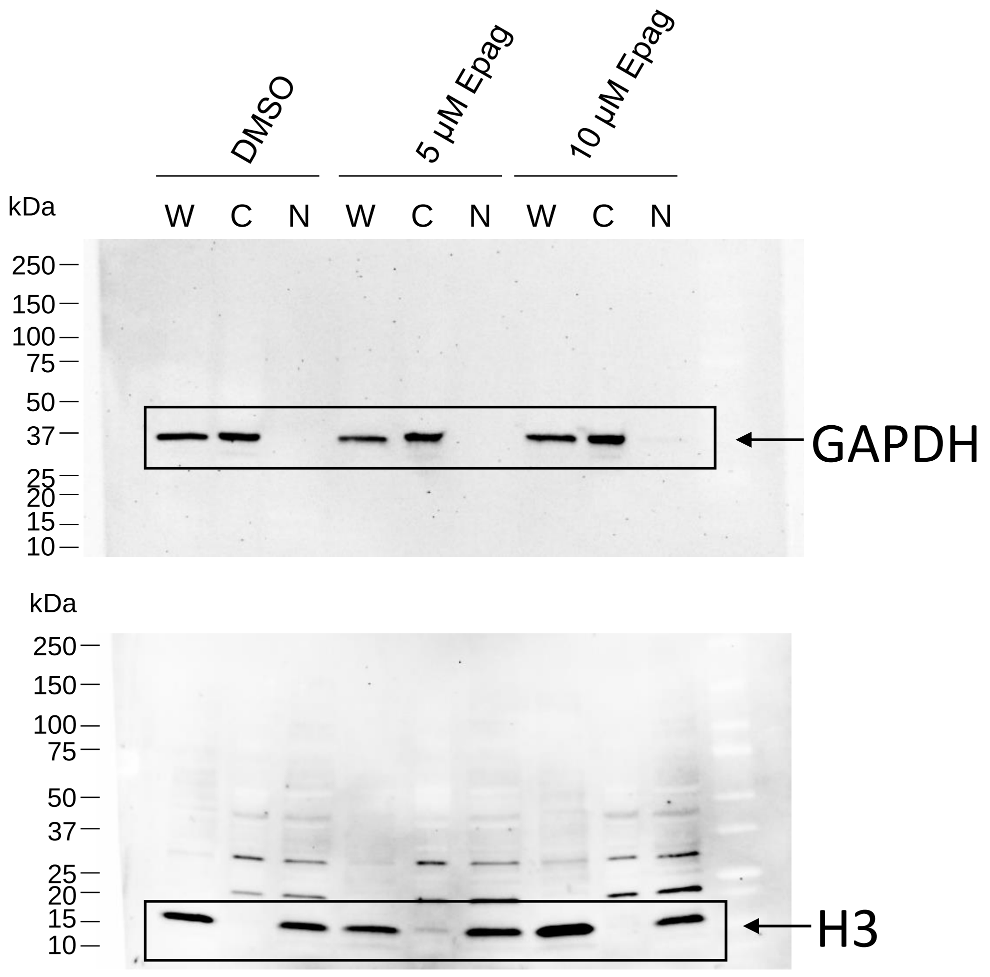

Full unedited blot for Figure 2C

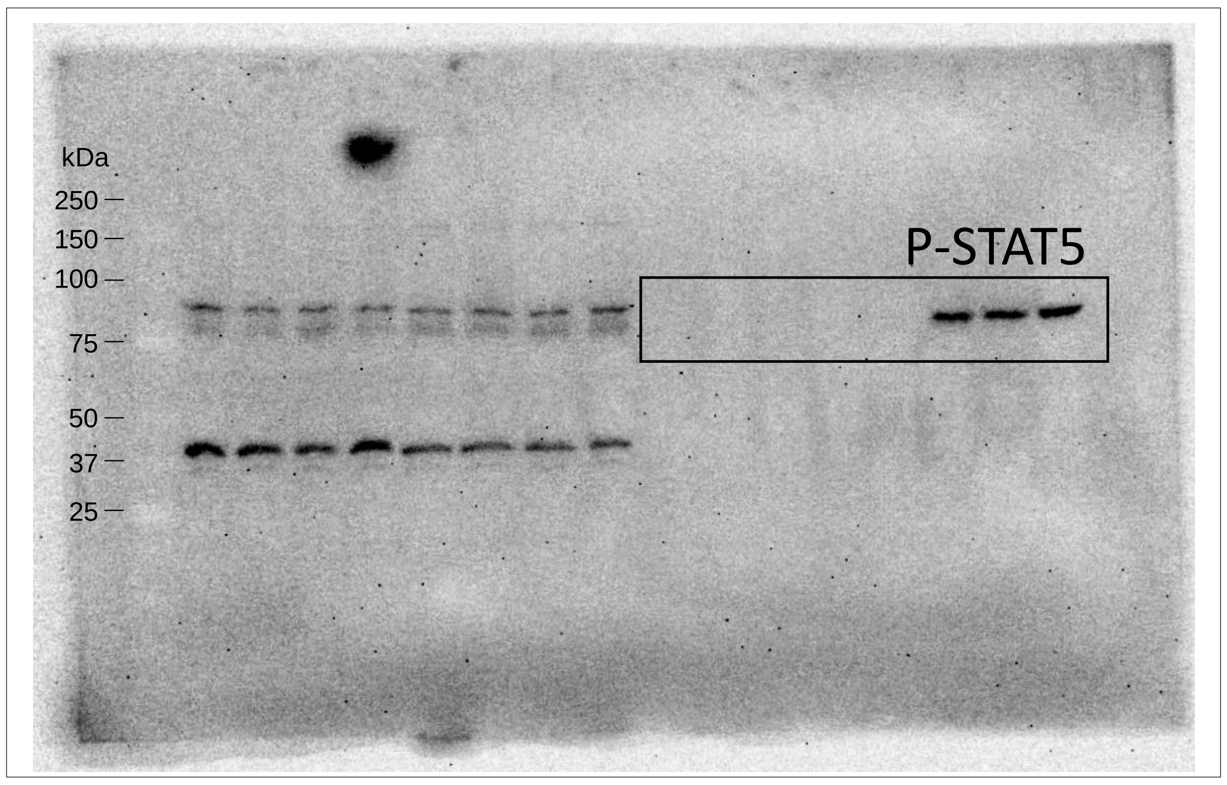

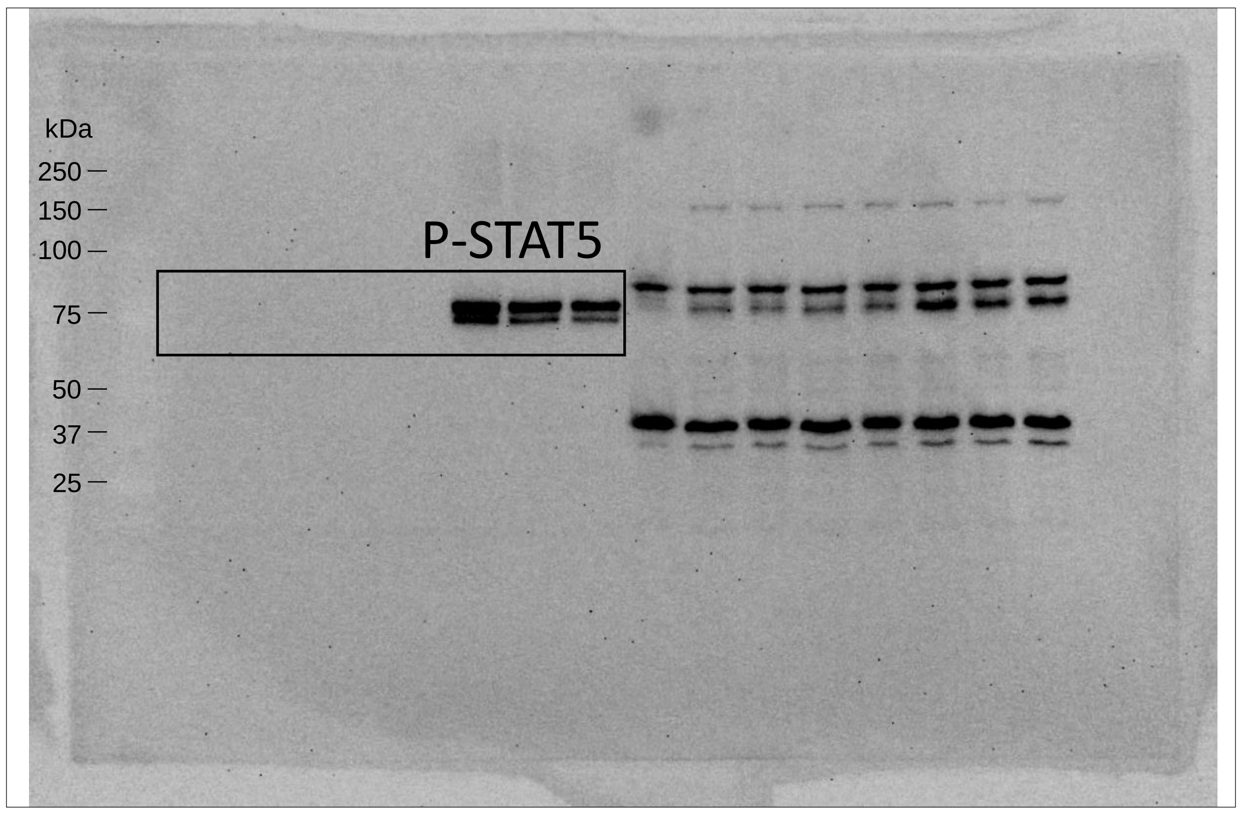

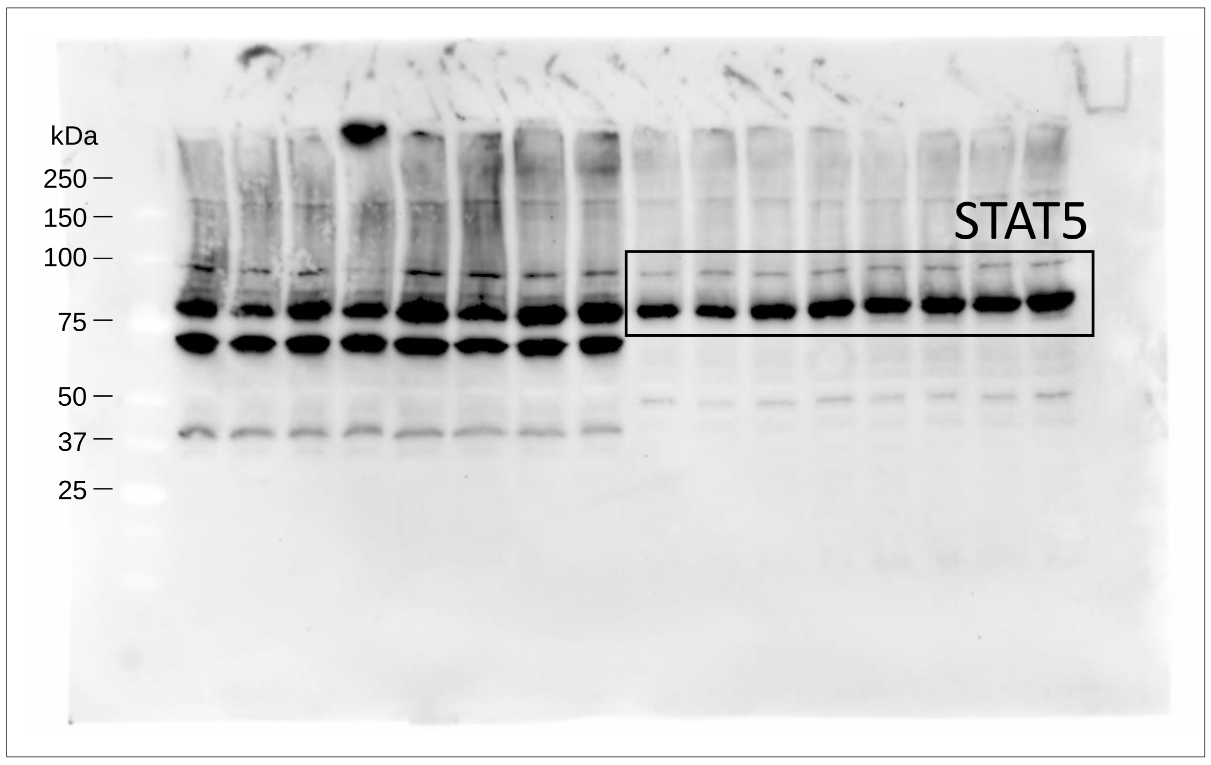

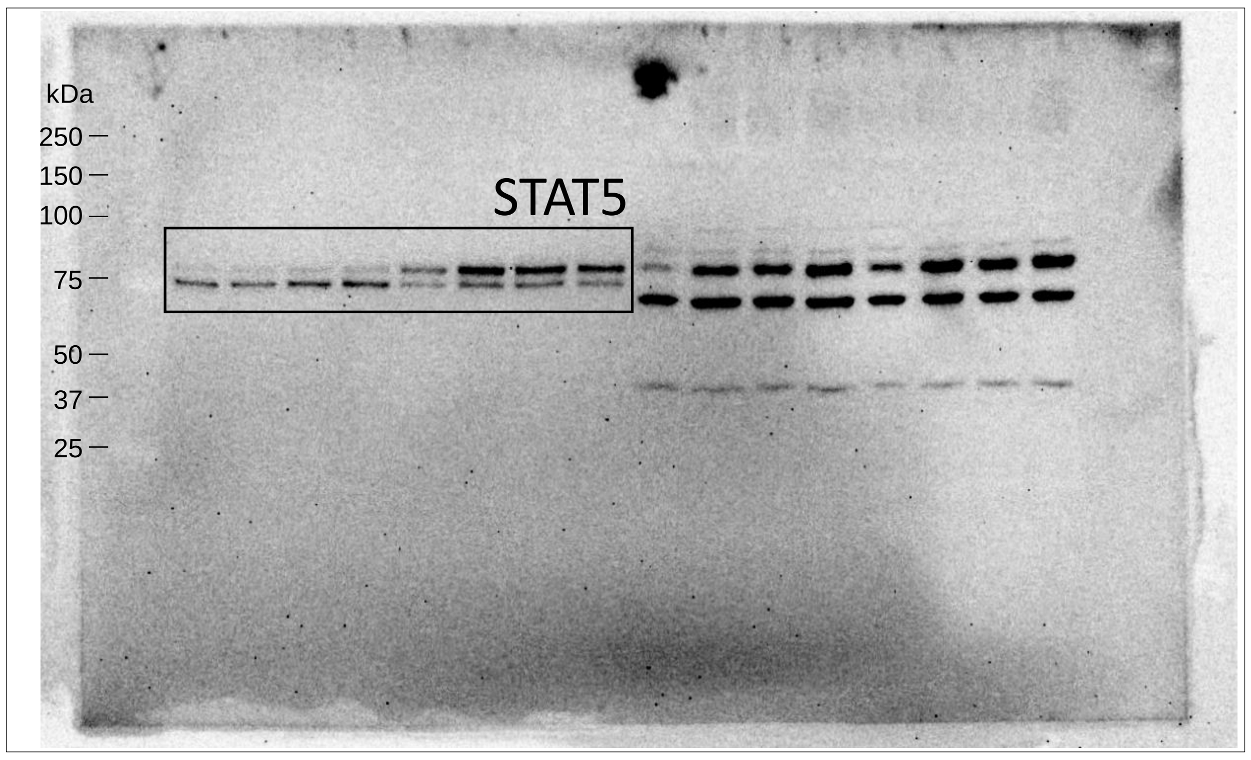

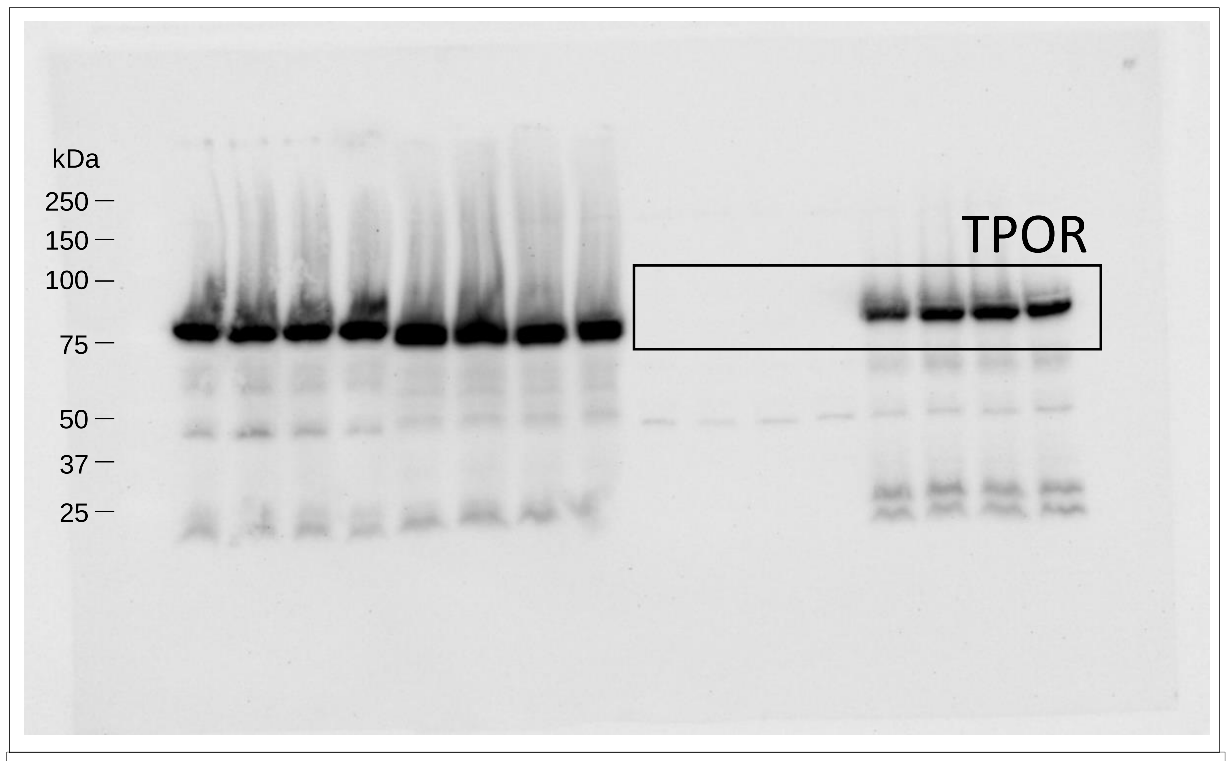

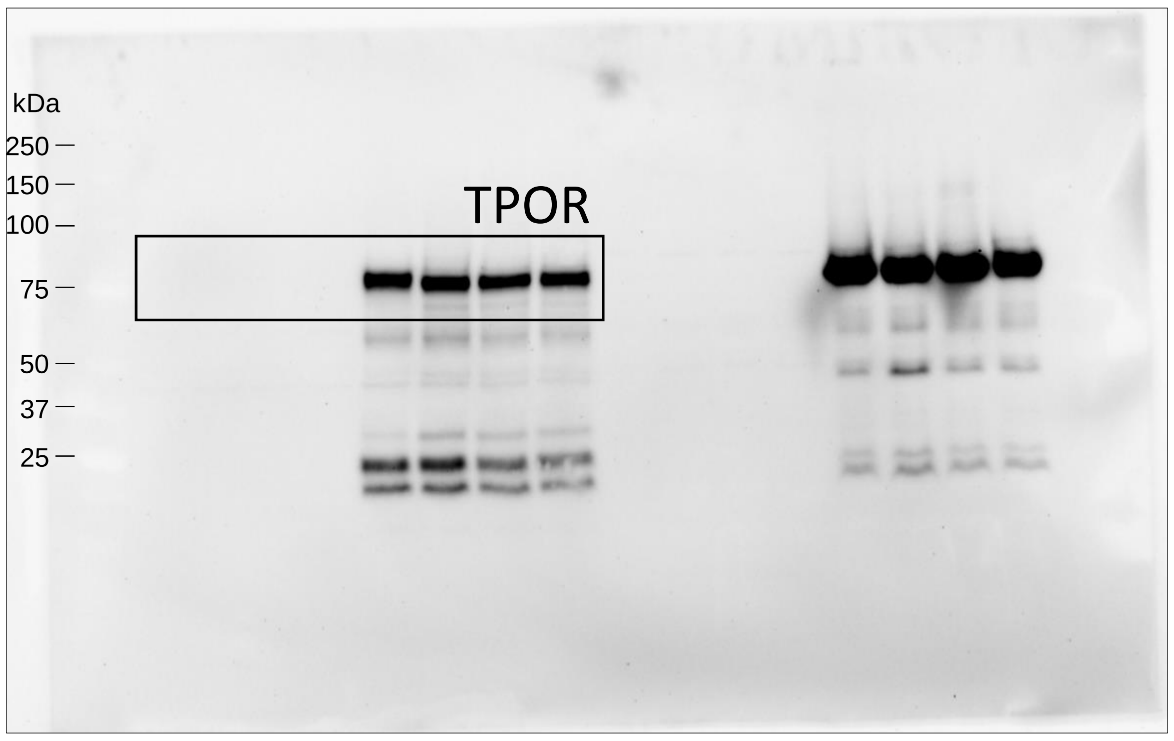

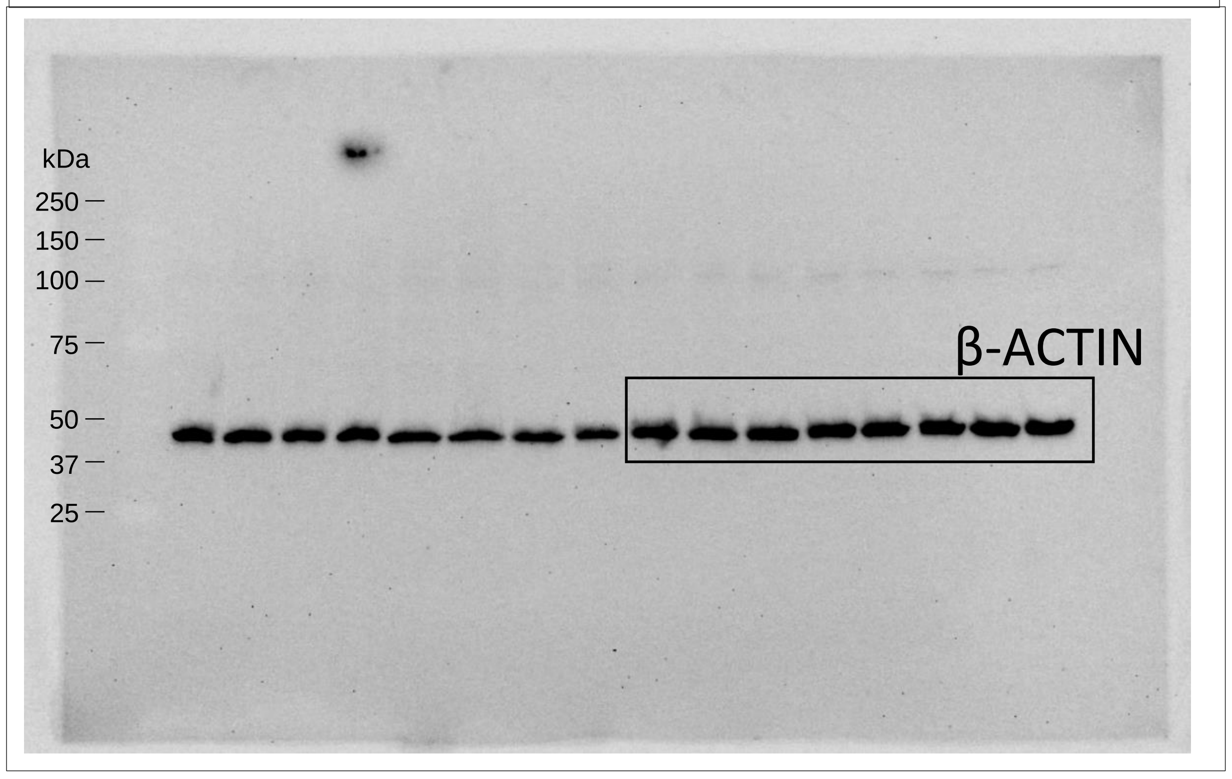

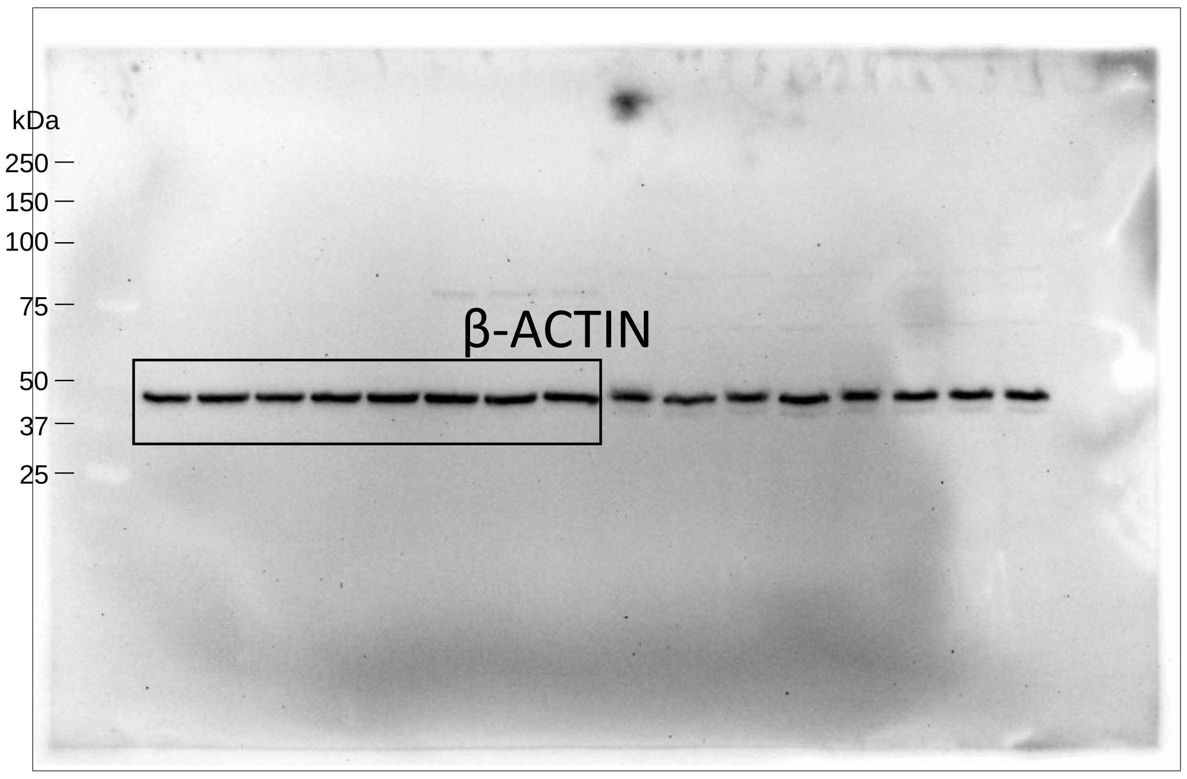

Full unedited blot for Figure 2D

5hmC

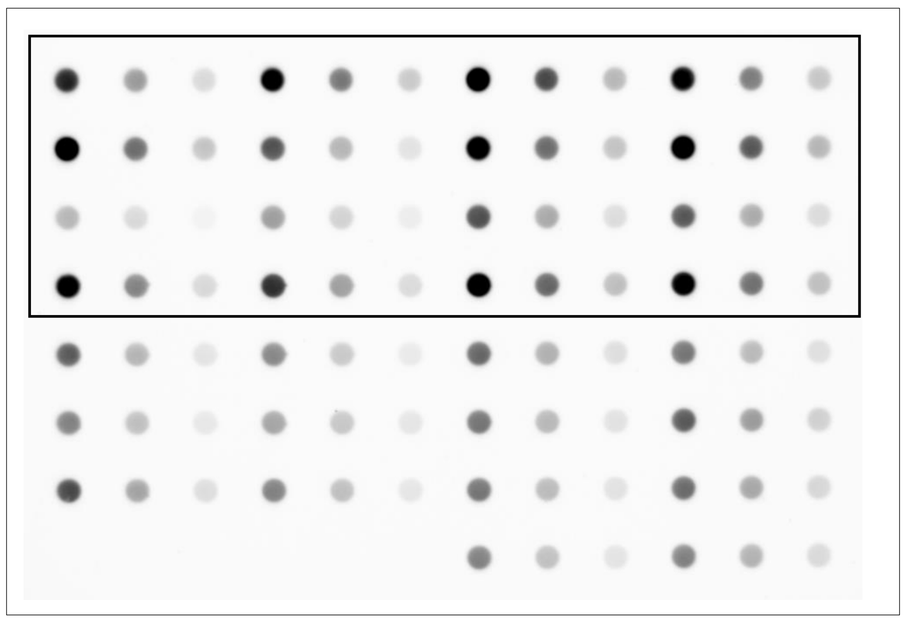

5mC

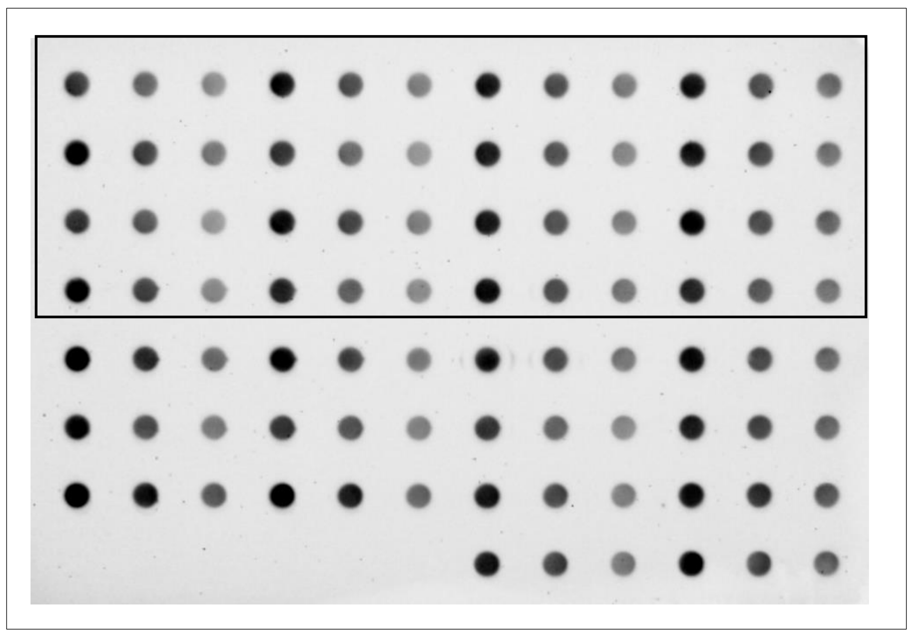

Loading

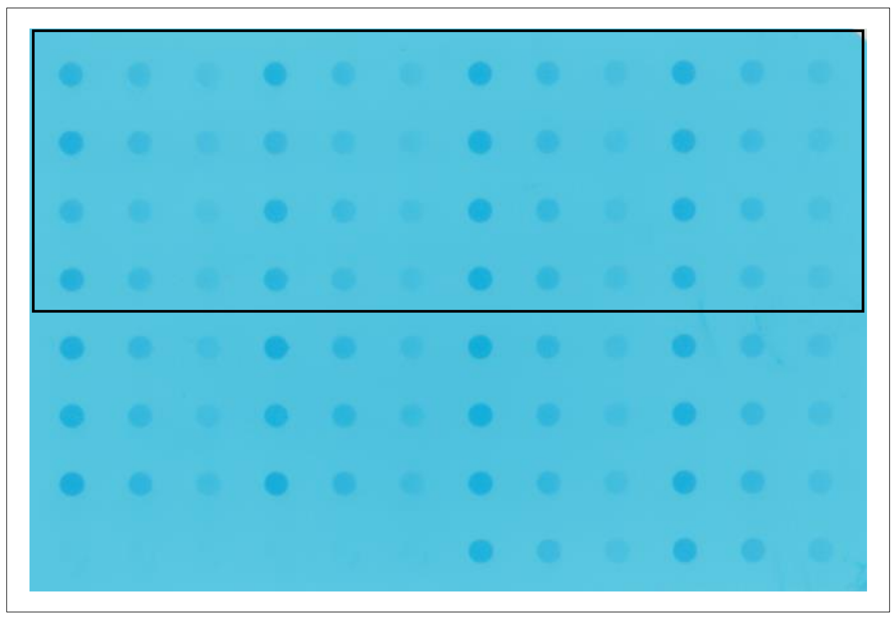

Full unedited blot for Figures 2F and 4A

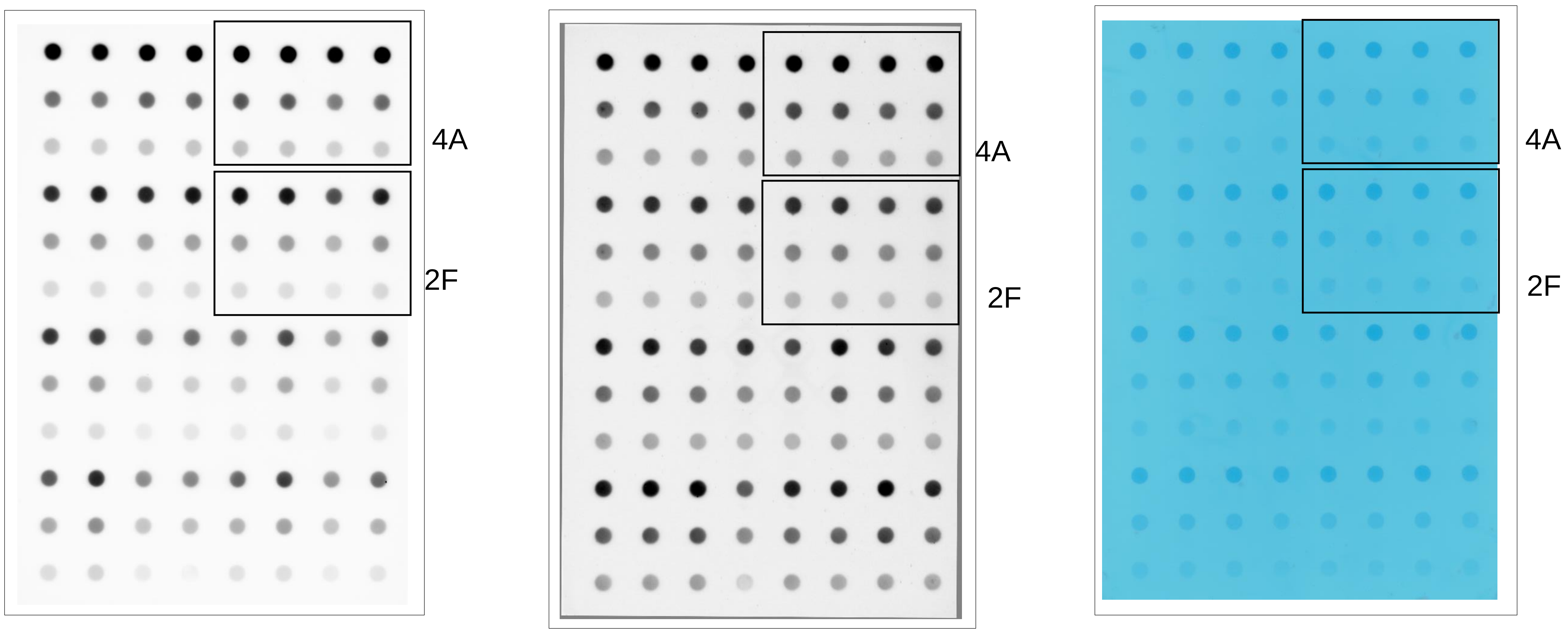

5hmC 5mC Loading
